# Total network controllability analysis discovers explainable drugs for Covid-19 treatment

**DOI:** 10.1186/s13062-023-00410-9

**Published:** 2023-09-05

**Authors:** Xinru Wei, Chunyu Pan, Xizhe Zhang, Weixiong Zhang

**Affiliations:** 1 Early Intervention Unit, Department of Psychiatry, The Affiliated Brain Hospital of Nanjing Medical University, Nanjing, Jiangsu 210029 China; 2 School of Biomedical Engineering and Informatics, Nanjing Medical University, Nanjing, Jiangsu 210001 China; 3 School of Computer Science and Engineering, Northeastern University, Shenyang, Liaoning 110167 China; 4 Department of Health Technology and Informatics, Department of Computing, The Hong Kong Polytechnic University, Hong Kong, China

**Keywords:** Total controllability, Explainable drugs for Covid-19, Control hubs

## Abstract

**Background:**

The active pursuit of network medicine for drug repurposing, particularly for combating Covid-19, has stimulated interest in the concept of structural controllability in cellular networks. We sought to extend this theory, focusing on the defense rather than control of the cell against viral infections. Accordingly, we extended structural controllability to total structural controllability and introduced the concept of control hubs. Perturbing any control hub may render the cell uncontrollable by exogenous stimuli like viral infections, so control hubs are ideal drug targets.

**Results:**

We developed an efficient algorithm to identify all control hubs, applying it to a largest homogeneous network of human protein interactions, including interactions between human and SARS-CoV-2 proteins. Our method recognized 65 druggable control hubs with enriched antiviral functions. Utilizing these hubs, we categorized potential drugs into four groups: antiviral and anti-inflammatory agents, drugs acting on the central nervous system, dietary supplements, and compounds enhancing immunity. An exemplification of our approach’s effectiveness, Fostamatinib, a drug initially developed for chronic immune thrombocytopenia, is now in clinical trials for treating Covid-19. Preclinical trial data demonstrated that Fostamatinib could reduce mortality rates, ICU stay length, and disease severity in Covid-19 patients.

**Conclusions:**

Our findings confirm the efficacy of our novel strategy that leverages control hubs as drug targets. This approach provides insights into the molecular mechanisms of potential therapeutics for Covid-19, making it a valuable tool for interpretable drug discovery. Our new approach is general and applicable to repurposing drugs for other diseases.

**Supplementary Information:**

The online version contains supplementary material available at 10.1186/s13062-023-00410-9.

## Key points

Extend network structural controllability to total network controllability, providing a practical method for identifying critical nodes that must be protected to maintain network controllability.

Extend and apply the new method to identifying drug targets in human protein-protein interaction networks.

Apply the new method to identify drugs that can be reused for Covid-19 treatment.

**Supplementary Information:**

The online version contains supplementary material available at 10.1186/s13062-023-00410-9.

## Background

The devastating Covid-19 pandemic caused by the severe acute respiratory syndrome coronavirus 2 (SARS-CoV-2) [1, 2] wreaked global havoc on all walks of life. SARS-CoV-2 and its variants have infected more than 767 million people and claimed more than 6.9 million lives worldwide, as reported to WHO (https://covid19.who.int; as of June 2023). The numbers are climbing despite several vaccines have been administrated in many countries. The viruses can penetrate the vaccines and spread rapidly in densely populated areas. Therefore, it is urgent to develop effective drugs for treating SARS-CoV-2 infection.

Drug discovery is notoriously costly and time-consuming [3], and developing new drugs for Covid-19 is challenging [4, 5]. One approach to shorten the period of finding effective medicine is to reposition or repurpose the drugs initially developed for other diseases, a major focus of drug discovery for Covid-19 [5–9]. However, the space for drug repurposing is enormous [9, 10]. The most popular computational approaches for drug repurposing take the perspective of systems biology or network medicine [10–16]. Among these are eminent methods based on the well-established network structural controllability [13–16]. Following the theory of structural controllability [17, 18], the cell is regarded as a network of genes/proteins that can be controlled by exogenous stimuli (e.g., viral infections or medical interventions) on a set of driver nodes (i.e., proteins) so that the cell can be driven from any state to the designated state in finite time. Structural controllability has been directly adopted to repurpose drugs for treating Covid-19 [15, 16]. Driver nodes targeted by existing drugs can give rise to putative reusable drugs, and the results were validated using bioinformatic methods and data in the literature [15, 16]. Structural controllability has been applied to protein-protein interaction networks [19, 20], gene regulatory networks [21], and metabolic networks [22]. The concept of driver nodes matches well with that of cancer driver genes [23] because frequent mutations (which are viewed as stimuli) in such genes may induce tumorigenesis, so the concepts of structural controllability and driver nodes have been applied to finding cancer driver genes as therapeutic targets for precision cancer treatment [14, 24]. Note that providing explainability is a prominent feature of drug repurposing approaches as it provides insight into the inner workings of the identified drugs and boosts confidence in adopting the drugs against the target disease [12].

While theoretically sound, straightforward application of structural controllability to drug repurposing is impractical. The key to the structural controllability of a network is a control scheme consisting of a set of control paths and their starting nodes (driver nodes or genes) that can be used to steer the network from any arbitrary state to the designated state in a finite time. Under this theory, a drug is used as an exogenous force to change the states of the cell, hopefully, from an infectious or cancerous state to a normal state. However, the normal states of a cell are typically unknown, so it remains unclear what external stimuli or drugs should be used. Moreover, the control scheme is not unique, and a control scheme typically has too many driver nodes to be practically manipulated at once to control the cell.

We pushed the envelope of the theory of structural controllability. Instead of attempting to control the cell, we aimed at protecting the cell from viral infections. Specifically, we expanded our perspective from structural controllability by a single network control scheme to a global view of total controllability over all control schemes for the network. We then introduced the concept of *control hubs*, which are nodes residing on a control path of every network control scheme. The control hubs are the most vulnerable spots to the structural controllability of the network; a perturbation to any control hub may render the network uncontrollable by any control scheme. Therefore, control hubs are ideal drug targets for protecting the cell from exogenous influences. Moreover, exploiting control hubs as drug targets is a more practical approach for drug repurposing because control hubs are typically an order less than driver nodes, as to be shown shortly. Without computing all control schemes, which is a #P-hard problem [25] (meaning no polynomial-time or efficient algorithm is known), we developed a polynomial-time (meaning efficient) algorithm for finding all control hubs for a network [26]. We applied our novel control hub-based drug repurposing approach to the largest homogenous human protein-protein interaction (PPI) network [27] (Table S1), along with the data of PPIs between SARS-CoV-2 and human [28, 29] (Table S2) and the data of drug targets [30] (Table S3), to discover control hubs that are targets of some existing drugs. Such druggable control hubs can not only be adopted for treating SARS-CoV-2 infections but also provide insights to explain the functions and mechanisms of the medicines in combatting the infection.

## Results

We first outline the rationale of our novel control-hub-based method and present its primary steps. We then apply it to an integrated network constructed using human and SARS-CoV-2 PPI data and the data of drugs and drug targets. We compare our new method with nine existing gene selection methods, including the structural-controllability-based driver-node method, to show its performance in finding drug targets for Covid-19. We then examine the 65 drug targets and the corresponding drugs identified by our new methods, using the data and results in the literature for validation.

### Total network controllability for drug repurposing

**Fig. 1:**
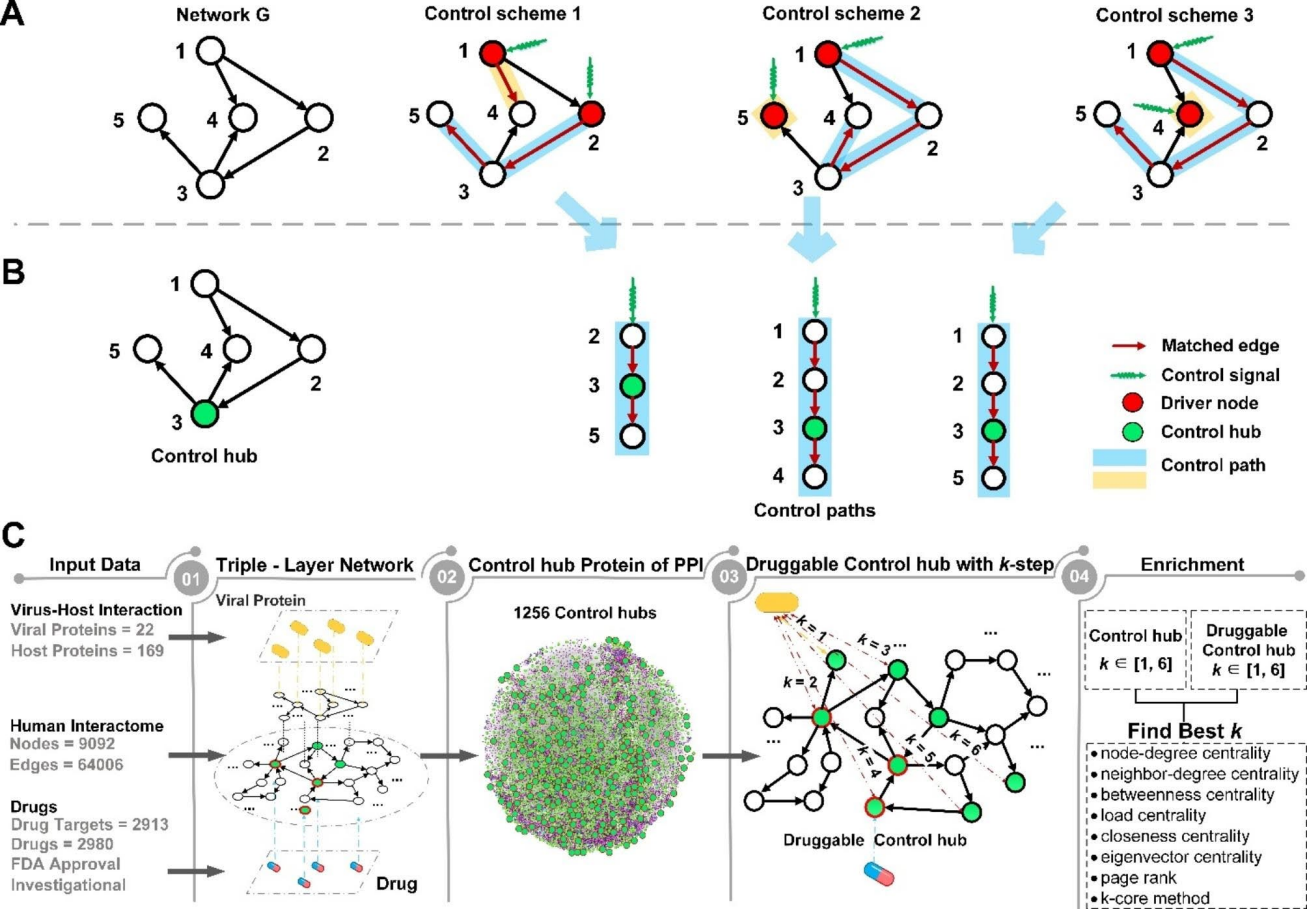
Control schemes and control hubs of a small network *G* and a new control-hub-based approach for drug repurposing. **(A)** Three distinct control schemes are identified by the maximum matching of *G*. Starting from a driver node (in red), a control path follows matched edges (in red). All control paths form a control scheme for *G*, and *G* has three control schemes. **(B)*** G* has one control hub node (in green), which appears in the middle of a control path of each control scheme. **(C)** The study design and the framework of a new control-hub-based approach. A triple-layer network connects the viral and human proteins and drugs and human protein targets. The study focused on the network community of proteins that were no more than two steps away from viral proteins (i.e., the 2-step community) and the 65 druggable control hubs within the community. The enrichment of druggable control hubs within the 2-step community was assessed against several gene ranking methods (see main text)

The primary concept of network structural controllability [17, 18] is a control scheme for a network. It consists of control paths such that every node in the network can be reached or controlled by the head node of the control path to which the node belongs (Fig. 1A). The head node is referred to as the driver or input node of the path. By exerting stimuli on the driver nodes, the network can be steered from any initial state to the designated state in a finite time. Structural controllability has been directly applied to repurposing drugs for treating Covid-19, where a small number of driver nodes targeted by drugs were used to find reusable drugs [15, 16].

However, driver nodes are a double-edged sword and can also be exploited by viruses to infect the cell. Viral infections are exogenous stimuli to the cell via the interactions of viral proteins and host receptors. These can transform cells from normal to abnormal to accommodate viral replication and propagation. During SARS-CoV-2 infection, the viral spike protein S engages human receptor angiotensin-converting enzyme 2 (ACE2) to enter the host cell, triggering a series of adverse signaling cascades [31].

Moreover, it is impractical to directly adopt structural controllability for controlling the cell or repurposing drugs. The control scheme is not unique (Fig. 1A). An exponential number of control schemes may exist, and one control scheme may have as many as half of the nodes in the network as driver nodes. For example, one control scheme for the human PPI network [27] (Table S1) contains 4,529 driver nodes, which are 49.8% of the 9,092 nodes in the network. Determining the most effective control scheme is a daunting task.

In light of these serious issues underlying the approaches to controlling the cell, we resorted to protecting the cell instead. We were motivated to identify critical genes, which, when perturbed, can render the cell uncontrollable by any control scheme or external stimulus on the driver nodes. Manipulating any of such critical genes can invalidate all the control schemes, so the cell is uncontrollable by undesired stimuli. To identify such critical genes, we extended structural controllability to *total controllability* by considering all control schemes and introducing a new concept of control hubs. A *control hub* is a middle node in one of the control paths of *every* control scheme (Fig. 1B). Blocking a control hub will block at least one control path of every control scheme, making the overall network uncontrollable.

Therefore, control hubs are ideal drug targets for protecting the cell from being manipulated by viral infections. If the genes that viruses act on are known, the control hubs close to these nodes can be chosen as designated drug targets to increase drug efficacy.

Since the concept of control hubs is built atop all control schemes, a technical obstacle is the potentially exponential number of control schemes for a network. Finding all control schemes using the current best method, i.e., maximum matching [32], is a computationally infeasible #P-complete problem [25]. To circumvent this difficulty, we developed an efficient, polynomial-time algorithm for finding all control hubs without computing all control schemes. The algorithm identified 1,256 control hubs in the human PPI network [27], which are 13.8% of all the 9,092 genes and 27.7% of the 4,529 driver genes for the network (Table S1).

Control hubs can act as surrogates to reusable drugs, i.e., we focus on those existing drugs that can target control hubs. While in theory, any drug-targeted control hubs can be used, the ones closer to exogenous stimuli (i.e., viral proteins) are preferred over the distant ones since blocking the former may prevent the spread of external influences sooner and more effectively.

### Finding drug targets for the treatment of viral infections

We capitalized on total controllability and control hubs and developed a drug-purposing method consisting of four major steps (Fig. 1C, see Methods and Supplemental Method S3). The first is constructing a network to integrate information on human PPI, virus PPI, drugs, and their targets. We used the largest homogenous human PPI network [27] (Table S1) and the data of PPIs between SARS-CoV-2 and human [28, 29] (Table S2) and the data of drug targets [30] (Table S3). The human PPI subnetwork and the virus PPI subnetwork are linked through the PPI between human and virus proteins, and the human PPI subnetwork and drug subnetwork are connected by the drug target information. The resulting network contains 9,092 nodes (proteins) from humans, 22 nodes from SARS-CoV-2, and 2,980 nodes of drugs. The overall network is relatively tight, with a total of 81,953 links.

The second step is to identify control hubs [26]. To focus on Covid-19, we left the technical details of our new method for finding control hubs to Methods and Supplemental Method S3. This control-hub finding method identified 1,256 control hubs in the network.

**Fig. 2:**
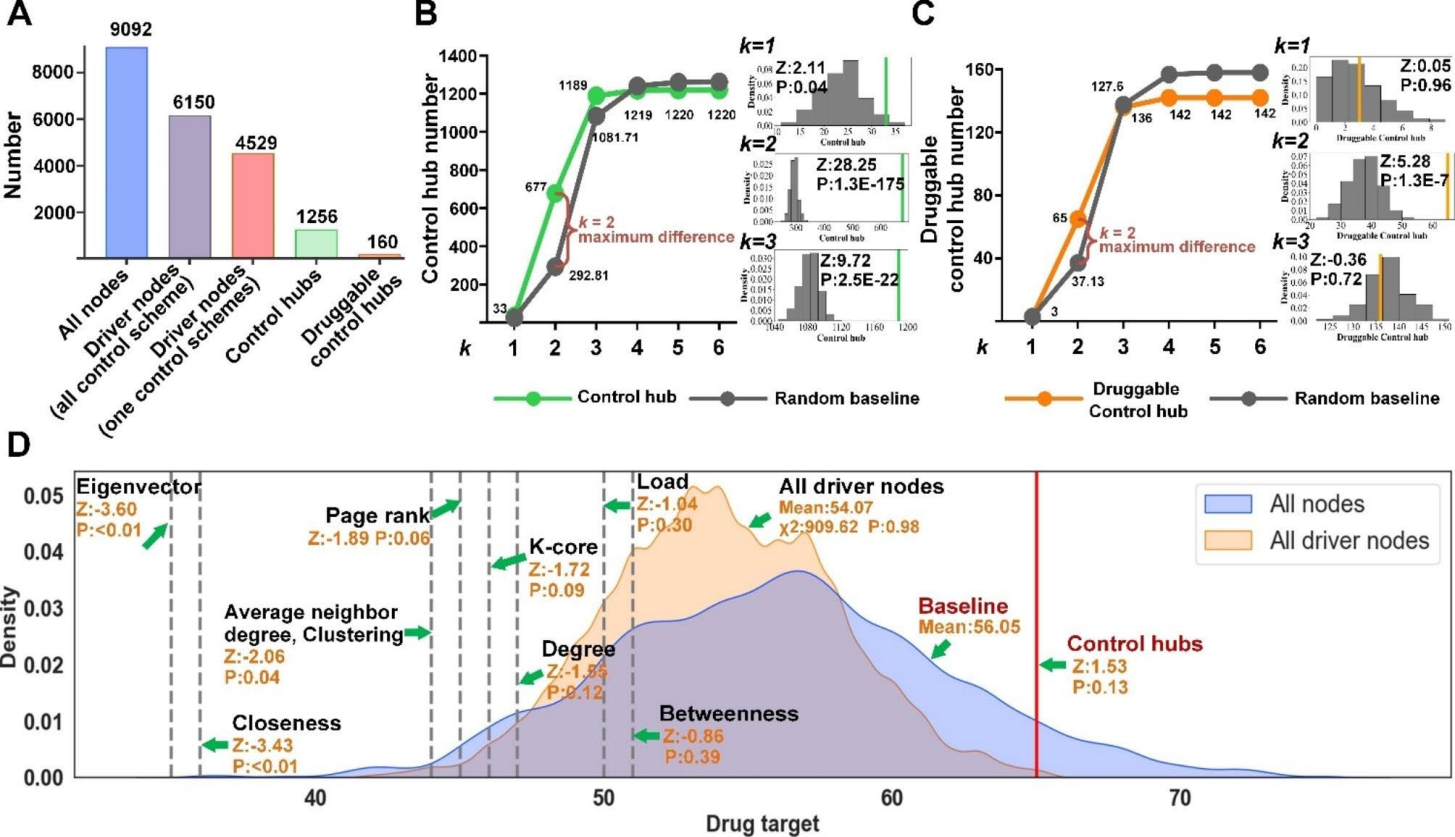
Comparison of druggable targets from different methods. **(A)** Distributions of the driver nodes, control hubs, and druggable control hubs in the human PPI network. **(B)** Determining that the 2-step community was most enriched with control hubs (the vertical axis) among all *k*-step communities of proteins with different *k* steps away from viral proteins (the horizontal axis). Statistical analysis was adopted to compare the number of control hubs (in green) within *k*-step communities against random empirical distributions (i.e., the baseline in grey). The three smaller figures on the side show random empirical distributions for *k* = 1, *k* = 2, and *k* = 3. The small figures include the values of enrichment of druggable targets (vertical green lines) by the new control-hub method. A z-test analysis showed that the highest increment of control hubs from the baseline occurred at *k* = 2. **(C)** The 2-step community was also enriched with druggable control hubs (the vertical axis). The same statistical analysis as in B) was performed. **(D)** Comparison of drug-target enrichment of the new method, the driver-node method, and the other eight node-ranking methods in the 2-step community

In the third step, to identify effective drug targets and drugs, we focused on the control hubs that were known targets of the existing drugs, categorically referred to as *druggable control hubs* hereafter. Among the 1,256 control hubs, 160 (12.7%) were drug targets (Fig. 2A).

Druggable control hubs were not equally effective for treating SARS-CoV-2 infection. Some control hubs may directly interact with viral proteins and thus are ideal drug targets, whereas many others are far away from viral proteins in the human PPI network (Fig. 1C). The closer a druggable control hub to virus proteins in the network, the more effective it should be for prohibiting viral infection.

Following this reasoning, in the fourth step, we examined the druggable control hubs in the community of proteins that were *k* steps away from the virus proteins in the PPI network, referred to as the *k*-step community, for convenience. A smaller *k* is preferred; the closer a control hub is to viral proteins, the more effective it is as a drug target to block viral infections. Two sets of enrichment tests, using the z-test, were performed to identify the best *k*-step community (see Methods). The first set of tests looked for the *k*-step community that was most enriched with control hubs among all *k*-step communities for different values of *k*, and the second set of tests assessed the enrichment of drug targets among the control hubs in the community chosen in the first test. The first z-test revealed that the 2-step community was most enriched with control hubs (z-score = 5.28, *p*-value = 1.3e^− 7^, Fig. 2B, S1A). It hosted 677 control hubs, among which 65 were drug targets (Table S4A). The second z-test confirmed that the 2-step community was also most enriched with druggable control hubs among all *k*-step communities (z-score = 28.25, *p*-value = 1.3e^− 175^, for *k* = 2, Fig. 2C, S1B).

In the last step, we assessed if our novel control-hub approach was the method of choice for finding drug targets. In particular, we compared it with nine existing methods, including the driver-node-based method and eight popular node ranking methods. These included node-degree centrality, neighbor-degree centrality, betweenness centrality, load centrality, closeness centrality, and eigenvector centrality, as well as Page-Rank, and k-core [32–42]. To facilitate the comparison and better understand these methods, we compared them against a statistical model of drug targets in the 2-step community. Assuming that any protein in the 2-step community was equally likely to be a drug target, the drug-target enrichment for 677 (i.e., the number of control hubs in the 2-step community) randomly selected proteins in the community should follow an empirical normal distribution (Fig. 2D). This empirical distribution was adopted as a statistical baseline model of drug-target enrichment. The enrichment of the 65 druggable control hubs in the 677 control hubs in the 2-step community substantially deviated from the baseline model (*z*-score = 1.53, *p*-value = 0.13; Fig. 2D). Likewise, the drug-target enrichment for 677 driver nodes randomly chosen from 965 driver nodes in the 2-step community should also obey an empirical normal distribution (Fig. 2D). The drug-target enrichment of our control-hub method was significantly better than the driver-node method’s (z-score = 2.82, *p*-value = 0.005). The driver-node method was slightly worse than the baseline model since the mean of the former was smaller than the mean of the latter (54.07 vs. 56.05; Fig. 2D), and the two distributions were statistically indistinguishable (*p*-value = 0.98, χ^2^-test; Fig. 2D). We measured the drug-target enrichments of the top 677 nodes from the eight gene-ranking methods. Unfortunately, these methods all underperformed; their z-tests against the random baseline model all resulted in negative z-scores (Fig. 2D). For instance, the Page-Rank method had a z-score=-1.89 with *p*-value = 0.06. This analysis showed that our novel control-hub method can identify the most drug targets and candidate drugs for Covid-19 treatment.

### Control hubs as drug targets for Covid-19 treatment

**Fig. 3:**
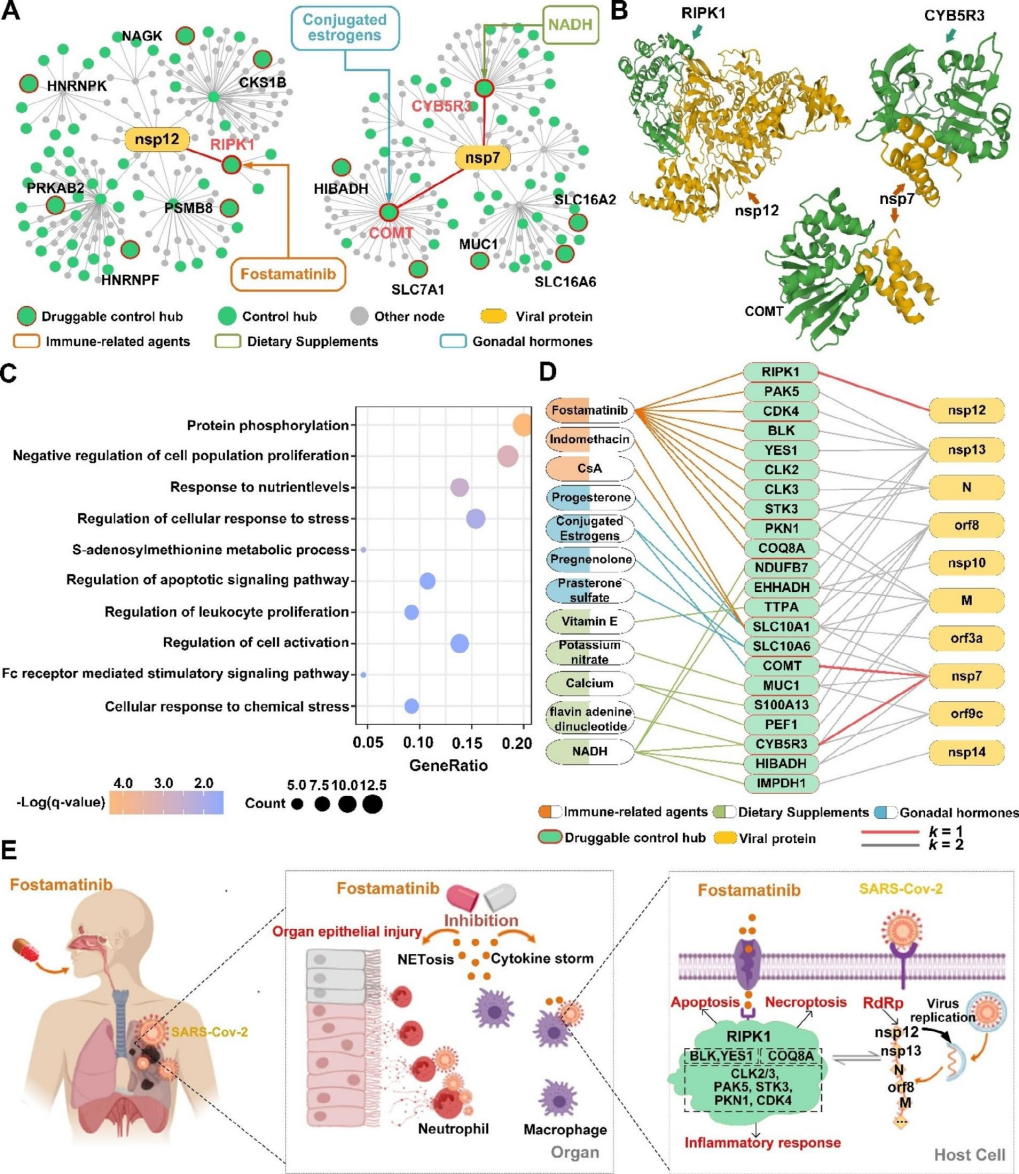
Potential therapeutic mechanisms of some druggable control hubs and selected drugs for treatment and/or prevention of Covid-19. **(A)** Network topologies of two SARS-Cov-2 proteins (nsp12 and nsp7 that are responsible for viral transcription and replication) and three human proteins (RIPK1, COMT, and CYB5R3) that directly interact with nsp12 and nsp7. **(B)** The binding structures of two SARS-Cov-2 proteins (nsp12 and nsp7) and three druggable control hubs (RIPK1, COMT, and CYB5R3). **(C)** The biological-process enrichment of the 65 druggable control hubs within the 2-step community, revealing their collective functions during viral infection. GeneRatio is the ratio between the number of observed proteins with a specific Go term and the total number of proteins of interest. **(D)** The interactions among SARS-Cov-2 proteins, key druggable control hubs, and drugs in three categories. Drugs are grouped based on their functions, marked in color. The drugs in orange correspond to immune-related agents, such as antineoplastic or Immunomodulating agents, in green are dietary supplements, such as Vitamins and Calcium; and in blue are gonadal hormones. **(E)** The potential therapeutic mechanisms of Fostamatinib for treating Covid-19. It reduces excessive immune and autoinflammatory responses by targeting ten control hubs, 9 of which are protein kinases and one on the p53 pathway

We examined the biological functions of the druggable control hubs to appreciate their role in SARS-Cov-2 infection and validate the new method using published results in the literature. Among all 160 druggable control hubs, three (RIPK1, CYB5R3, and COMT) directly interact with nonstructural proteins of SARS-CoV-2 [28, 29] (Fig. 3A and B, S2; Table 1, S4A). RIPK1 can bind with viral nonstructural protein nsp12 [28, 29], the RNA-dependent RNA polymerase (RdRp) of SARS-CoV-2 [43] (Fig. 3A and B). nsp12 promotes viral replication and inhibits the host’s innate immune response by suppressing the activity of interferon regulatory factor 3 (IRF3), which is key to interferon production [44]. Both CYB5R3 and COMT interact with the nsp7 protein of SARS-CoV-2 (Fig. 3A and B), which forms a tetramer with viral nsp8 [45] and functions as a cofactor of the viral RdRp, nsp12 [43]. Since nsp12 and nsp7 are essential for viral transcription and replication, blocking the interactions of RIPK1 with nsp12, CYB5R3 with nsp7, and COMT with nsp7 can potentially inhibit or suppress viral replication.

**Table 1:** Twenty-eight of the 65 druggable control hubs (in Table S4) within no more than two steps away from SARS-Cov-2 proteins in the triple-layer PPI network. Shown are the druggable control hub (the **Host Protein** column), engaging **Viral Protein** (and the [**Distance**] between the host and viral proteins), **Host Protein Function**, and **Targeting Drugs** (and the **Total number** of drugs targeting the protein). Drugs are grouped based on their function categories marked in color. Drugs in orange correspond to immune-related agents, in green are dietary supplements, and in blue are gonadal hormones. The seven druggable control hubs discussed in the text are marked in grey. At least two drugs target the rest 21 druggable control hubs

Host Protein	Viral Protein [Distance]	Host Protein Function	Targeting Drugs (Total number)
RIPK1	nsp12 [1]	Inflammation, cell death, pathogen recognition	Fostamatinib
CYB5R3	nsp7 [1], orf9c [2], M [2]	Cholesterol biosynthetic process.	NADH, Flavin adenine dinucleotide, Copper
COMT	nsp7 [1]	Neurotransmitter catabolic process	Conjugated estrogens, Diethylstilbestrol, Tolcapone (15)
SLC10A1	nsp7 [2], orf8 [2], orf3a [2], M [2]	Bile acid and bile salt transport	Conjugated estrogens, Progesterone, Indomethacin, Cyclosporine A (CsA) (18)
SLC10A6	nsp7 [2], orf8 [2]	Transmembrane transport	Pregnenolone, Prasterone sulfate
MUC1	nsp7 [2], orf9c [2], orf8 [2]	Forming protective mucous barriers on epithelial surfaces	Fostamatinib, Potassium nitrate, TG4010
TTPA	orf8 [2], nsp13 [2]	Vitamin E metabolic process	Vitamin E supplements (6)
OPRM1	orf8 [2]	A class of opioid receptors	Tramadol, Morphine, Codeine (47)
TSPO	orf8 [2]	Steroid hormone synthesis, immune response	Lorazepam, Temazepam, Alprazolam (12)
GLUL	orf8 [2]	Ammonia and glutamate detoxification, acid-base homeostasis	Pegvisomant, L-Glutamine, Methionine (8)
IMPDH1	nsp14 [2]	Regulate cell growth	NADH, Ribavirin, Mycophenolate mofetil (7)
S100A13	orf8 [2]	Cell cycle progression and differentiation	Calcium (7)
RARA	orf8 [2]	Regulation of differentiation of clock genes.	Adapalene, Acitretin, Alitretinoin (7)
MAPK1	nsp13 [2]	Differentiation, transcription regulation	Isoprenaline, Arsenic trioxide (6)
SLC16A2	nsp7 [2]	Transporter of thyroid hormone	Pyruvic acid, Tyrosine, L-Leucine (6)
CDK4	nsp13 [2]	Cell cycle progression	Fostamatinib, Palbociclib, Ribociclib, Abemaciclib
HDAC4	nsp13 [2]	Transcriptional regulation, cell cycle progression	Zinc, Romidepsin (4)
PEF1	M [2]	Response to calcium ion	Calcium (3)
CATSPER1	M [2]	Calcium ion transport	Calcium (3)
GNMT	orf8 [2]	Methionine metabolic process	Ademetionine, Glycine, Citric acid
PCYT1A	orf8 [2]	Regulation of phosphatidylcholine biosynthesis.	Choline, Lamivudine, Choline salicylate
SLC7A1	nsp7 [2]	Involved in amino acid transport.	L-Lysine, L-Arginine, Ornithine
YES1	nsp13 [2]	Innate immune response; transmembrane receptor protein	Fostamatinib, Dasatinib
ASPH	orf8 [2]	Calcium ion transmembrane transport	Aspartic acid, Succinic acid
MAT2A	nsp9 [2]	Catalyzes the production of AdoMet	Ademetionine, Methionine
PCNA	nsp15 [2]	Positive regulation of DNA repair	Liothyronine, Acetylsalicylic acid
SRR	nsp15 [2]	Catalyzes the synthesis of D-serine from L-serine.	Pyridoxal phosphate, Serine
SULT2B1	nsp8 [2]	Catalyze sulfate conjugation of many hormones, and drugs.	Prasterone, Pregnenolone

RIPK1 encodes serine/threonine-protein kinase 1, plays a role in necroptosis, apoptosis, and inflammatory response, and mediates cell death and inflammation [46]. SARS-CoV-2 infection promotes the expression of RIPK1 in the lung of Covid-19 patients, and small-molecule inhibitors of RIPK1 can reduce the viral load of SARS-CoV-2 and proinflammatory cytokines in human lung organoids, indicating that the virus hijacks RIPK1-mediated immune response for its replication and propagation [47]. RIPK1 is targeted by Fostamatinib (Table 1, S4A; Fig. 3A), a drug under intense scrutiny for treating SARS-CoV-2 infection [47–53]. Fostamatinib is an inhibitor of spleen tyrosine kinase originally approved for treating chronic immune thrombocytopenia. Fostamatinib is effective in a mouse model of acute lung injury and acute respiratory syndrome, symptoms observed in Covid-19 patients [49]. A clinical trial with a small sample of hospitalized Covid-19 patients (30 with fostamatinib versus 29 with placebo) showed that Fostamatinib could lower mortality, shorten the length of ICU stay, and reduce the disease severity of critically ill patients [50].

CYB5R3 encodes NADH-cytochrome B5 reductase 3, a flavoprotein with oxidation functions. It is targeted by three drugs (Table 1, S4A), two of which (NADH and Flavin adenine dinucleotide) are under clinical investigation for Covid-19 treatment. NADH is an energy booster for treating chronic fatigue syndrome and improving high blood pressure and jet lag, among many other symptoms. NADH, i.e., nicotinamide adenine dinucleotide (NAD) + hydrogen (H), is the central catalyst of cellular metabolism, a chemical naturally produced in humans and plays a role in ATP production. The SARS-CoV-2 genome does not encode enzymes for ATP generation, and the virus needs to hijack host functions for viral synthesis and assembly. Therefore, NAD is a battlefield for viral infection and host immunity [54]. Indeed, coronavirus infection dysregulates the NAD metabolome, as indicated in a preclinical study [55]. Moreover, early phases 2 and 3 clinical trials showed that medication of NADH in a mixture of two metabolic activators could significantly shorten the time to complete recovery of SARS-CoV-2 infection [56].

COMT encodes catechol-O-methyltransferase that can degrade estrogens, catecholamines, and neurotransmitters such as dopamine, epinephrine, and norepinephrine. It is targeted by 14 FDA-approved drugs, including Conjugated estrogens (Table 1, S4A). Conjugated estrogens are a mixture of estrogen hormones for treating hypoestrogenism-related symptoms. Estrogen has been indicated as a susceptibility factor of SARS-CoV-2 infection [57], as women are less susceptible to Covid-19 [58, 59] and mice with weaker estrogen receptor signaling due to respiratory coronavirus infection exhibit increased morbidity and mortality [60].

Beyond the three druggable control hubs that directly interact with viral proteins, 19 druggable control hubs in the 2-step community engage more than one viral protein via another protein, and four of them (SLC10A1, SLC10A6, MUC1, and TTPA) are targeted by more than one drug (Table 1, S4A; Fig. S2). The potential of these four druggable control hubs for Covid-19 treatment is discussed in Supplemental Result S1.

In short, the 65 druggable control hubs within the 2-step community were enriched with biological functions related to cell (particularly leukocyte) proliferation, cellular response to (chemical) stress, regulation of apoptotic signaling, and response to nutrient levels (Fig. 3C). All these results combined revealed the essential roles these control hubs might play in prohibiting the replication and proliferation of SARS-CoV-2. The results also revealed the essential immune-related signaling pathways induced by the virus and paved the way for understanding and explaining the therapeutic mechanisms of the drugs for Covid-19 treatment.

### Drugs for the treatment of SARS-CoV-2 infection

The 65 druggable control hubs within the 2-step community were targeted by 185 existing drugs (Table 2, S5; Fig. 3D). As of June 2022, 38 were under clinical trials (https://clinicaltrials.gov/ct2/home). It is desirable to use drugs with multiple targets to gain treatment efficacy; the potency of a drug can be estimated by the number of control hubs it targets. Remarkably, 15 drugs target more than one control hub, and seven target more than two druggable control hubs (Table 2, S5).

**Table 2:** Drugs for the treatment and/or prevention of Covid-19. Fifteen candidate drugs target more than one druggable control hub, among which two belong to immune-related agents (in orange), nine are dietary supplements (in green), and two are gonadal hormones (in blue). The drugs with “*” are under clinical trial for treating Covid-19. Detailed information is available in Table S5

Drug	Control hub Number	Control hub
Fostamatinib*	10	COQ8A, CLK3, CLK2, YES1, BLK, PAK5, STK3, RIPK1, PKN1, CDK4
NADH*	5	CYB5R3, EHHADH, HIBADH, NDUFB7, IMPDH1
Calcium phosphate dihydrate	3	S100A13, PEF1, CATSPER1
Calcium Citrate	3	S100A13, PEF1, CATSPER1
Calcium Phosphate	3	S100A13, PEF1, CATSPER1
Conjugated estrogens*	2	SLC10A1, COMT
Progesterone*	2	SLC10A1, SULT2B1
Phenethyl Isothiocyanate	5	HNRNPF, TPM1,
Ademetionine	3	COMT, GNMT, MAT2A
Copper	2	AHCY, CYB5R3
Aspartic acid	2	ASPH, ACY3
Methionine	2	GLUL, MAT2A
Citric acid	2	HGS, GNMT
Liothyronine	2	PCNA, SLC10A1
Liotrix	2	SLC10A1, SLC16A2

Among the seven drugs targeting more than two control hubs were Fostamatinib, NADH, and three dietary calcium supplements (Table 2, S5). Fostamatinib is in phase 3 clinical trial after a promising phase 2 trial for Covid-19 treatment [61]. Experimental and clinical data showed that Fostamatinib inhibits neutrophil extracellular traps (NETs), which entrap and eliminate pathogens during viral and bacterial infections and may cause adverse injury to surrounding tissues by themselves or by increasing proinflammatory responses [62] (Fig. 3E). Activation and overreaction of innate and adaptive inflammatory responses during SARS-Cov-2 infection induce NETs, contributing to immunothrombosis in acute respiratory distress syndrome (ARDS) commonly seen in Covid-19 patients [48, 62–65]. Moreover, coherent antiviral therapeutic functions of Fostamatinib emerged after examining the functions of the control hubs that the drug targets (Fig. 3D and E; Table S5). Among the ten control hubs that Fostamatinib targets, 7 (RIPK1, CLK2, CLK3, PAK5, STK3, PKN1, and CDK4) are serine/threonine type protein kinases, and two (BLK and YES1) encode Src family tyrosine kinases, all of which play essential roles in cell proliferation, cell differentiation, and programmed cell death [66]. CLK2 and CLK3 encode members of the serine/threonine type protein kinase family, and PAK5, STK3, PKN1, and CDK4 encode, respectively, one of the three members of the group II PAK family of serine/threonine kinases, serine/threonine-protein kinase 3, serine/threonine protein kinase N, and cyclin-dependent serine/threonine kinase. Plus, RIPK1 encodes receptor-interacting serine/threonine-protein kinase 1 and directly interacts with the viral RdRp nsp12, as discussed earlier. Interestingly, while not being a kinase, the remaining target COQ8A encodes a mitochondrial protein functioning in an electron-transferring membrane protein complex in the respiratory chain. Its expression is induced by the tumor suppressor p53 in response to DNA damage, and inhibition of its expression suppresses p53-induced apoptosis. Combined, the inhibitory function on NETs and kinase functions of 9 of the ten control hubs targeted by Fostamatinib suggested it to be potent for Covid-19 treatment by acting broadly on components of autoimmune, tumor repression, and inflammatory viral response pathways (Fig. 3E).

NADH targets five control hubs, including CYB5R3, NDUFB7, IMPDH1, EHHADH, and HIBADH. CYB5R3, encoding NADH-cytochrome B5 reductase 3, directly interacts with nsp7, a cofactor of the viral RdRp. Blocking the interactions of CYB5R3 with nsp7 could be a mechanism to suppress viral replication, as previously discussed. NDUFB7, a component of the mitochondrial respiratory chain, connects to virus-induced mitochondrial dysfunction and may be targeted to inhibit this pathway [67]. IMPDH1, associated with guanine nucleotide synthesis, also presents another possible drug target, as its inhibition could reduce the guanine nucleotide pool, potentially decreasing SARS-CoV-2 synthesis efficiency [68, 69]. The roles of EHHADH and HIBADH in SARS-CoV-2, both implicated in metabolic processes, are yet to be elucidated and warrant further study. On the other hand, recent findings that highlight a decrease in NADH levels in Covid-19 patients [54], along with the observed alterations in the NAD metabolome [55], further underscore the prospective therapeutic implications of NADH in attenuating the consequences of viral infection.

Three dietary calcium supplements, Calcium Citrate, Calcium Phosphate, and Calcium phosphate dihydrate, target control hubs S100A13, PEF1, and CATSPER1. These calcium-binding proteins are essential in various cellular functions, possibly associated with viral infections, including SARS-CoV-2. However, existing literature does not substantiate a direct connection between these proteins and SARS-CoV-2 infection mechanisms. Conversely, several clinical studies have identified low serum calcium levels as significant prognostic factors influencing the mortality, severity, and comorbidity of SARS-CoV-2 infections [69–72]. As of a note, six pharmaceutical agents related to vitamin E target the control hub TTPA (encoding a soluble protein that is a form of vitamin E) and are now in clinical trials for Covid-19 treatment (Table 1, S5). The precise relationship between TTPA and SARS-CoV-2 infection remains unclear. These results indicated that calcium, vitamin E, and many other micronutrients should be adopted as adjuvant therapy against viral infection.

In summary, the repurposed drugs fall into four major categories (Table S5), (1) antiviral and anti-inflammatory agents that are subscribed for virus infection and cancer treatment, (2) dietary supplements including NADH and Calcium that boost human immunity, (3) hormones, including conjugated estrogens, and (4) drugs acting on central nerve systems. Combined, the medicines in the first three categories help boost immunity to overcome viral infections’ adverse stress and influence.

## Discussion

Network medicine for drug repurposing has gained popularity and momentum since the Covid-19 pandemic [10–16]. Most of these network-biology methods hinge upon the idea that important proteins can be surrogates for identifying medicines. However, these methods operate under different notions of what constitutes important proteins in biological networks. For example, proteins with high degrees of connectivity may be considered essential since they supposedly affect many neighboring proteins.

Network structural controllability [17, 18] has been adopted as an approach to network medicine. Using driver nodes as drug targets is particularly appealing for Covid-19 drug repurposing [15, 16]. However, while theoretically sound, this approach is impractical for drug repurposing, as discussed earlier. Our drug-target enrichment analysis showed that such a direct application of structural controllability was no better than random selection (Fig. 2D).

Our most important contributions are the extension of structural controllability to total controllability and the new perspective of protecting rather than controlling cells. In particular, we were motivated to protect the cell from any exogenous stimulus, particularly viral infections, because this is relatively easier and more effective than controlling the cell. Methodologically, by extending structural controllability to total controllability, we introduced control hubs to identify the critical spots in the cell that were important for the controllability of the cell. We used targeting drugs as external influences to make the cell uncontrollable by any viral infection. Therefore, control hubs are an effective vehicle for drug repurposing, as demonstrated in the current study. It is not coincidental that many control hubs are also targets of existing drugs, as shown in our drug-target enrichment analysis (Fig. 2D). Instead, the result revealed that proteins with biological importance, particularly those related to immunity, resided in critical positions in the human PPI network.

To treat or prevent viral infections, control hubs in the human PPI network should be protected by blocking their interactions with viral proteins or interactions with one another, which can prevent or curtail the spread of viral influence. Control hubs are thus excellent candidate drug targets for treating and preventing Covid-19. Identifying such drug targets was entirely data-driven and used no information on gene functions. The information on drug targets from DrugBank was brought to the analysis at a late stage of drug repurposing. We used highly confident homogenous human and SARS-CoV-2 PPI data from HEK293T cells under well-controlled conditions [26–29] to avoid possible false-positive results from heterogeneous data.

Most viral proteins interacting with human proteins are nonstructural, and many of them are responsible for viral transcription and replication as well as suppression of the innate and adaptive immune responses of the host (Table S4, S7). Many druggable control hubs have immunity and antiviral functions such as regulating apoptotic signaling, cellular response to stress, leukocyte proliferation, and cell population proliferation (Fig. 3C; Table 1, S4). Nutrient levels are another key factor these control hubs responded to (Fig. 3C; Table 1, S4). Identifying these druggable control hubs has shed light on potential therapeutic mechanisms for Covid-19 treatment (Fig. 3E; Table 2, S5), thereby enhancing our novel, explainable method for drug repurposing [12]. For example, RIPK1 interacts with viral RdRp nsp12, and CYB5R3 and COMT interact with nsp7, a cofactor of viral RdRp (Fig. 3A and B). RIPK1 is targeted by Fostamatinib, CYB5R3 by three drugs, including NADH, and COMT by 15 drugs including Conjugated estrogens (Table 2, S4A, S5). Although the precise connection between control hub proteins and SARS-CoV-2 infection is yet to be examined, the existing experimental and clinical data support the efficacy of these drugs in blocking or suppressing SARS-CoV-2’s transcription or replication, thereby bolstering host immunity.

An interesting result is identifying Fostamatinib as a Covid-19 drug, particularly suitable for hospitalized patients (Fig. 3E; Table 2, S5). This drug is currently in clinical trials for Covid-19. Identifying Fostamatinib and other drugs for Covid-19 treatment proves the concept of control hubs as drug targets and firmly validates our novel control hub-based approach. Moreover, the functions of the ten control hubs targeted by Fostamatinib explain well the mechanistic mode of action that the medicine may perform and its biological functions in treating severely ill Covid-19 patients. It is encouraging that this data-driven result was supported by the experimental results on a mouse model of acute lung injury and acute respiratory syndrome [49] and the data of a preliminary clinical trial of critically ill patients [61]. Altogether, the biological functions and experimental data suggested that the drug prevents exaggerated autoinflammatory immune responses68,69 and alleviates the burden of cytokine storms [73, 74] in severe Covid-19 cases.

A substantial number of control hubs in the 2-step community of the human PPI network are not targets of any existing drug. These control hubs, particularly the membrane proteins that function on the NF-κB pathway (Fig. S4), can be used to propose testable hypotheses for new drug development for Covid-19 therapy.

The novel approach presented here can be extended to repurpose drugs for various other diseases, which we will pursue in future research. It is also worth mentioning that we exploited and deployed the novel concepts of total controllability and control hubs to develop a new concept of cancer-keeper genes as therapeutic targets for cancer treatment [75]. Some cancer-keeper genes were experimentally validated in vitro in cancer cell lines and in vivo in a mouse bladder cancer model [75].

## Conclusions

Our study presents a novel approach to drug repurposing with significant implications for COVID-19 treatment and beyond. Focusing on control hubs as drug targets can potentially unlock a new strategy for combating current and future viral threats. Furthermore, many control hubs we identified not yet targeted by existing drugs present exciting avenues for developing new antiviral medications.

## Methods

### An overview of the novel control-hub-based method for drug repurposing

The new method consists of the following four major steps discussed in the subsequent subsections.

1. Construction of a biological network. In the current study, an integrated network of human PPI, virus PPI, drug targets, and drugs;
2. Identification of control hubs [26]; the algorithmic details are in Supplemental Method S3.
3. Determination of the k-step network community with nodes k steps away from the viral proteins and enriched with drug targets;
4. Assessment and validation of the new method by comparison with nine existing gene selection methods, including the structural-controllability-based driver-node method.

### Construction of a triple-layer interaction network from viruses to humans to drugs

The central layer of the network contained the human protein-protein interaction (PPI) network that was constructed using the human Huri-Union binary protein interaction dataset [27]. This is the largest homogenous human protein interactome with data collected primarily from HEK923T cells and validated in multiple orthogonal assays. The network consists of 9,092 nodes or proteins and 64,006 interactions (Table S1).

The SARS-CoV-2 AP-MS data26 from HEK293T cells was added to include the layer of viral proteins. The dataset contains 332 high-confidence virus-host interactions between 27 SARS-CoV-2 proteins and 332 human proteins, which were used to link the human and virus PPI subnetworks. Since the human PPI network contains only 9,092 proteins, the final triple-layer network contains 169 interactions between 22 viral and 169 human proteins (Table S2). The 3D Structural Interactome between SARS-CoV-2 and host proteins was retrieved from SARS-CoV-2-Human Interactome Browser [76].

The network was further expanded to include the layer of drugs and their human protein targets using the data from DrugBank [30]. The links between drugs and their protein targets were used to link the human PPI subnetwork and drug subnetwork. We only included drugs approved by FDA and under investigation. The drug-target interactome contains 17,780 interactions between 2,981 drugs and 2,914 target proteins (Table S3). The information on drug categories in DrugBank was used to group drugs (Table S5).

### Identification of control hubs

A network can be controlled by exerting control signals on *driver nodes* [17, 29] (Fig. 1A). To analyze the controllability of a network, maximum matching from graph theory was adopted to find the minimum set of driver nodes [18, 77]. A *maximum matching* is the maximum set of edges that do not share nodes in common [32]. The edges of maximum matching form paths of the network, which start from *head nodes* and along the matching edges, reach tail nodes. The head nodes of a maximum matching are taken as driver nodes, and the paths are *control paths* [78] (Fig. 1A), constituting a *control scheme*. The maximum matching is not unique for most networks, and neither is the control scheme (Fig. 1A).

A node may occupy distinct positions – a driver, a tail, or a middle node – in control paths of different control schemes. Some nodes may always remain as middle nodes in all control schemes, and such nodes are defined as *control hub*s (Fig. 1B). All control hubs can be identified in polynomial time without computing all control schemes [26]; the algorithmic details are available in our previous work [26, 79, 80].

### Identification of druggable control hubs within k-step from viral proteins and candidate drugs

A breadth-first traversal of the triple-layer PPI network was carried out to find the reachable control hubs within no more than k steps from some viral proteins. The traversal started from the viral proteins and ignored edge directions. The process terminated after all nodes at *k* steps from the beginning were visited.

All control hubs encountered in the process of the breadth-first traversal were reported. These control hubs were further checked against DrugBank [30] to identify druggable control hubs.

The best value of *k* for the *k*-step community was determined by two z-tests, as described in the main text, along with the statistical significance of the two-tailed p-value. The z-tests were done using the following formulas:

$$\frac{{D}_{F} - mean\left({S}_{F}\right)}{SD of {S}_{F}}$$

where ${D}_{F}$ is the number of nodes in the *k*-step community overlapping with druggable control hubs or control hubs, $mean\left({S}_{F}\right)$ is the average number of druggable control hubs or control hubs overlapping with a random set of nodes of the same size as the *k*-step community, and $SD of {S}_{F}$ is the standard deviation of ${S}_{F}$ from 1,000 randomly chosen sets of nodes in the community. The details are in Supplemental Method S2.

### Node ranking methods

Nine popular node ranking methods were used to compare with the new control-hub-based method. These include two methods related to node degree (degree centrality [33] and average neighbor degree [34]), three related to network shortest paths (betweenness centrality [35], load centrality [36, 37], and closeness centrality [38]), three related to network structures (eigenvector centrality [39, 40], clustering coefficient [81], and K-core [42]), and a classical web ranking algorithm (page rank [82, 83]). A detailed description of these ranking algorithms is in network [84] and Supplemental Method S1.

### Feature enrichment analysis

See Supplemental Method S2 for details.

### Gene enrichment analysis

To explore the biological processes in which the 65 druggable control hubs were involved, functional annotation analyses with Kyoto Encyclopedia of Genes and Genomes (KEGG) pathway annotation and Gene Ontology (GO) annotation were performed using Metascape [85]. The Go biological process terms and KEGG pathways with FDR-corrected *p*-value < 0.05 were reported.

### Electronic supplementary material

Below is the link to the electronic supplementary material.

Supplementary Material 1

Supplementary Material 2

Supplementary Material 3

## Data Availability

The datasets used in the study are in Tables S1-S3. The datasets and software of our method are freely available on GitHub at https://github.com/network-control-lab/control-hubs.

## References

[1] Gorbalenya AE, Baker SC, Baric RS, de Groot RJ, Drosten C, Gulyaeva AA, Haagmans BL, Lauber C, Leontovich AM, Neuman BW (2020). The species severe acute respiratory syndrome-related coronavirus: classifying 2019-nCoV and naming it SARS-CoV-2. Nat Microbiol.

[2] Chen G, Wu D, Guo W, Cao Y, Huang D, Wang H, Wang T, Zhang X, Chen H, Yu H (2020). Clinical and immunological features of severe and moderate coronavirus disease 2019. J Clin Invest.

[3] Beck A, Goetsch L, Dumontet C, Corvaïa N (2017). Strategies and challenges for the next generation of antibody-drug conjugates. Nat Rev Drug Discov.

[4] Abd El-Aziz TM, Stockand JD (2020). Recent progress and challenges in drug development against COVID-19 coronavirus (SARS-CoV-2)-an update on the status. Infect Genet Evol.

[5] Kim S (2022). COVID-19 Drug Development. J Microbiol Biotechnol.

[6] Riva L, Yuan SF, Yin X, Martin-Sancho L, Matsunaga N, Pache L, Burgstaller-Muehlbacher S, De Jesus PD, Teriete P, Hull MV (2020). Discovery of SARS-CoV-2 antiviral drugs through large-scale compound repurposing. Nature.

[7] Dotolo S, Marabotti A, Facchiano A, Tagliaferri R (2021). A review on drug repurposing applicable to COVID-19. Brief Bioinform.

[8] Chakraborty C, Sharma AR, Bhattacharya M, Agoramoorthy G, Lee SS (2021). The Drug Repurposing for COVID-19 clinical trials provide very effective therapeutic combinations: Lessons learned from Major Clinical Studies. Front Pharmacol.

[9] Ng YL, Salim CK, Chu JJH (2021). Drug repurposing for COVID-19: approaches, challenges and promising candidates. Pharmacol Ther.

[10] Pushpakom S, Iorio F, Eyers PA, Escott KJ, Hopper S, Wells A, Doig A, Guilliams T, Latimer J, McNamee C (2019). Drug repurposing: progress, challenges and recommendations. Nat Rev Drug Discov.

[11] Sadegh S, Matschinske J, Blumenthal DB, Galindez G, Kacprowski T, List M, Nasirigerdeh R, Oubounyt M, Pichlmair A, Rose TD (2020). Exploring the SARS-CoV-2 virus-host-drug interactome for drug repurposing. Nat Commun.

[12] Jimenez-Luna J, Grisoni F, Schneider G (2020). Drug discovery with explainable artificial intelligence. Nat Mach Intell.

[13] Morselli Gysi D, do Valle Í, Zitnik M, Ameli A, Gan X, Varol O, Ghiassian SD, Patten JJ, Davey RA, Loscalzo J et al. (2021). Network medicine framework for identifying drug-repurposing opportunities for COVID-19. Proc Natl Acad Sci 118. 10.1073/pnas.2025581118 PMC8126852 33906951

[14] Guo WF, Zhang SW, Zeng T, Akutsu T, Chen L (2020). Network control principles for identifying personalized driver genes in cancer. Brief Bioinform.

[15] Ackerman EE, Shoemaker JE (2020). Network Controllability-Based prioritization of candidates for SARS-CoV-2 Drug Repositioning. Viruses.

[16] Siminea N, Popescu V, Martin S, Florea JA, Gavril D, Gheorghe G, Iţcuş AM, Kanhaiya C, Pacioglu K, Popa O et al. L.L. (2022). Network analytics for drug repurposing in COVID-19. *Brief Bioinform* 23. 10.1093/bib/bbab490 PMC8690228 34864885

[17] Lin C-T (1974). Structural controllability. IEEE Trans Autom Control.

[18] Liu YY, Slotine JJ, Barabási AL (2011). Controllability of complex networks. Nature.

[19] Zhang X (2018). Altering Indispensable Proteins in Controlling Directed human protein Interaction Network. IEEE/ACM Trans Comput Biol Bioinform.

[20] Pan C, Zhu Y, Yu M, Zhao Y, Zhang C, Zhang X, Yao Y (2021). Control analysis of protein-protein Interaction Network reveals potential Regulatory targets for MYCN. Front Oncol.

[21] Qian X, Ivanov I, Ghaffari N, Dougherty ER (2009). Intervention in gene regulatory networks via greedy control policies based on long-run behavior. BMC Syst Biol.

[22] Asgari Y, Salehzadeh-Yazdi A, Schreiber F, Masoudi-Nejad A (2013). Controllability in cancer metabolic networks according to drug targets as driver nodes. PLoS ONE.

[23] Bailey MH, Tokheim C, Porta-Pardo E, Sengupta S, Bertrand D, Weerasinghe A, Colaprico A, Wendl MC, Kim J, Reardon B (2018). Comprehensive characterization of Cancer driver genes and mutations. Cell.

[24] Guo WF, Zhang SW, Feng YH, Liang J, Zeng T, Chen L (2021). Network controllability-based algorithm to target personalized driver genes for discovering combinatorial drugs of individual patients. Nucleic Acids Res.

[25] Valiant LG (1979). The complexity of computing the permanent. Theor Comput Sci.

[26] Zhang X, Pan C, Zhang W. (2022). Control hubs of complex networks and a polynomial-time identification algorithm. arXiv:2206.01188. 10.48550/arXiv.2206.01188.

[27] Luck K, Kim DK, Lambourne L, Spirohn K, Begg BE, Bian W, Brignall R, Cafarelli T, Campos-Laborie FJ, Charloteaux B (2020). A reference map of the human binary protein interactome. Nature.

[28] Gordon DE, Jang GM, Bouhaddou M, Xu J, Obernier K, White KM, O’Meara MJ, Rezelj VV, Guo JZ, Swaney DL (2020). A SARS-CoV-2 protein interaction map reveals targets for drug repurposing. Nature.

[29] Gordon DE, Hiatt J, Bouhaddou M, Rezelj VV, Ulferts S, Braberg H, Jureka AS, Obernier K, Guo JZ, Batra J et al. (2020). Comparative host-coronavirus protein interaction networks reveal pan-viral disease mechanisms. Science 370. 10.1126/science.abe9403 PMC7808408 33060197

[30] Wishart DS, Knox C, Guo AC, Cheng D, Shrivastava S, Tzur D, Gautam B, Hassanali M (2008). DrugBank: a knowledgebase for drugs, drug actions and drug targets. Nucleic Acids Res.

[31] Hoffmann M, Kleine-Weber H, Schroeder S, Krüger N, Herrler T, Erichsen S, Schiergens TS, Herrler G, Wu NH, Nitsche A (2020). SARS-CoV-2 cell entry depends on ACE2 and TMPRSS2 and is blocked by a clinically proven protease inhibitor. Cell.

[32] Hopcroft JE, Karp RM (1973). An n^5/2 algorithm for maximum matchings in bipartite graphs. SIAM J Comput.

[33] Borgatti SP, Halgin DS. Analyzing affiliation networks. Volume 1. SAGE Publications Ltd); 2011.

[34] Barrat A, Barthelemy M, Pastor-Satorras R, Vespignani A (2004). The architecture of complex weighted networks. Proc Natl Acad Sci.

[35] Brandes U (2008). On variants of shortest-path betweenness centrality and their generic computation. Soc Networks.

[36] Newman ME (2001). Scientific collaboration networks. II. Shortest paths, weighted networks, and centrality. Phys Rev E.

[37] Goh K-I, Kahng B, Kim D (2001). Universal behavior of load distribution in scale-free networks. Phys Rev Lett.

[38] Freeman LC (1978). Centrality in social networks conceptual clarification. Soc Networks.

[39] Bonacich P (1987). Power and centrality: a family of measures. Am J Sociol.

[40] Newman ME. Networks—An introduction. Oxford University Press; 2012.

[41] Zhang JX, Chen DB, Dong Q, Zhao Z-D (2016). Identifying a set of influential spreaders in complex networks. Sci Rep.

[42] Batagelj V, Zaversnik M. (2003). An O (m) algorithm for cores decomposition of networks. *arXiv preprint cs/0310049*. 10.48550/arXiv.cs/0310049.

[43] Gao Y, Yan L, Huang Y, Liu F, Zhao Y, Cao L, Wang T, Sun Q, Ming Z, Zhang L (2020). Structure of the RNA-dependent RNA polymerase from COVID-19 virus. Science.

[44] Wang W, Zhou Z, Xiao X, Tian Z, Dong X, Wang C, Li L, Ren L, Lei X, Xiang Z (2021). SARS-CoV-2 nsp12 attenuates type I interferon production by inhibiting IRF3 nuclear translocation. Cell Mol Immunol.

[45] Zhang C, Li L, He J, Chen C, Su D (2021). Nonstructural protein 7 and 8 complexes of SARS-CoV-2. Protein Sci.

[46] Mifflin L, Ofengeim D, Yuan J (2020). Receptor-interacting protein kinase 1 (RIPK1) as a therapeutic target. Nat Rev Drug Discov.

[47] Xu G, Li Y, Zhang S, Peng H, Wang Y, Li D, Jin T, He Z, Tong Y, Qi C (2021). SARS-CoV-2 promotes RIPK1 activation to facilitate viral propagation. Cell Res.

[48] Strich JR, Ramos-Benitez MJ, Randazzo D, Stein SR, Babyak A, Davey RT, Suffredini AF, Childs RW, Chertow DS (2021). Fostamatinib inhibits neutrophils Extracellular Traps Induced by COVID-19 patient plasma: a potential therapeutic. J Infect Dis.

[49] Kost-Alimova M, Sidhom EH, Satyam A, Chamberlain BT, Dvela-Levitt M, Melanson M, Alper SL, Santos J, Gutierrez J, Subramanian A (2020). A high-content screen for mucin-1-Reducing compounds identifies Fostamatinib as a candidate for Rapid Repurposing for Acute Lung Injury. Cell Rep Med.

[50] Strich JR, Tian X, Samour M, King CS, Shlobin O, Reger R, Cohen J, Ahmad K, Brown AW, Khangoora V (2022). Fostamatinib for the treatment of hospitalized adults with Coronavirus Disease 2019: a Randomized Trial. Clin Infect Dis.

[51] Hoepel W, Chen HJ, Geyer CE, Allahverdiyeva S, Manz XD, de Taeye SW, Aman J, Mes L, Steenhuis M, Griffith GR et al. (2021). High titers and low fucosylation of early human anti-SARS-CoV-2 IgG promote inflammation by alveolar macrophages. Sci Transl Med 13. 10.1126/scitranslmed.abf8654 PMC8158960 33979301

[52] Apostolidis SA, Sarkar A, Giannini HM, Goel RR, Mathew D, Suzuki A, Baxter AE, Greenplate AR, Alanio C, Abdel-Hakeem M et al. (2022). Signaling through FcγRIIA and the C5a-C5aR pathway mediate platelet hyperactivation in COVID-19. Front Immunol 13. 10.3389/fimmu.2022.834988 PMC8928747 35309299

[53] Rivero-García I, Castresana-Aguirre M, Guglielmo L, Guala D, Sonnhammer ELL (2021). Drug repurposing improves disease targeting 11-fold and can be augmented by network module targeting, applied to COVID-19. Sci Rep.

[54] Brenner C (2022). Viral infection as an NAD(+) battlefield. Nat Metab.

[55] Heer CD, Sanderson DJ, Voth LS, Alhammad YMO, Schmidt MS, Trammell SAJ, Perlman S, Cohen MS, Fehr AR, Brenner C (2020). Coronavirus infection and PARP expression dysregulate the NAD metabolome: an actionable component of innate immunity. J Biol Chem.

[56] Altay O, Arif M, Li XY, Yang H, Aydin M, Alkurt G, Kim W, Akyol D, Zhang C, Dinler-Doganay G et al. (2021). Combined metabolic activators accelerates recovery in mild-to-moderate COVID-19. Adv Sci 8. 10.1002/advs.202101222 PMC8420376 34180141

[57] Brandi ML (2022). Are sex hormones promising candidates to explain sex disparities in the COVID-19 pandemic?. Rev Endocr Metab Disord.

[58] Chen N, Zhou M, Dong X, Qu J, Gong F, Han Y, Qiu Y, Wang J, Liu Y, Wei Y (2020). Epidemiological and clinical characteristics of 99 cases of 2019 novel coronavirus pneumonia in Wuhan, China: a descriptive study. Lancet.

[59] Guan WJ, Ni ZY, Hu Y, Liang WH, Ou CQ, He JX, Liu L, Shan H, Lei CL, Hui DSC (2020). Clinical characteristics of Coronavirus Disease 2019 in China. N Engl J Med.

[60] Channappanavar R, Fett C, Mack M, Eyck T, Meyerholz PP, Perlman S (2017). Sex-based differences in susceptibility to severe Acute Respiratory Syndrome Coronavirus infection. J Immunol.

[61] Strich JR, Tian X, Samour M, King CS, Shlobin O, Reger R, Cohen J, Ahmad K, Brown AW, Khangoora V (2021). Fostamatinib for the treatment of hospitalized adults with COVD-19 a randomized trial. Clin Infect Dis.

[62] Mutua V, Gershwin LJ (2021). A review of Neutrophil Extracellular Traps (NETs) in Disease: potential Anti-NETs therapeutics. Clin Rev Allergy Immunol.

[63] Middleton EA, He XY, Denorme F, Campbell RA, Ng D, Salvatore SP, Mostyka M, Baxter-Stoltzfus A, Borczuk AC, Loda M (2020). Neutrophil extracellular traps contribute to immunothrombosis in COVID-19 acute respiratory distress syndrome. Blood.

[64] Bautista-Becerril B, Campi-Caballero R, Sevilla-Fuentes S, Hernández-Regino LM, Hanono A, Flores-Bustamante A, González-Flores J, García-Ávila CA, Aquino-Gálvez A et al. Castillejos-López, M. (2021). Immunothrombosis in COVID-19: Implications of Neutrophil Extracellular Traps. *Biomolecules* 11. 10.3390/biom11050694 PMC8148218 34066385

[65] Szturmowicz M, Demkow U (2021). Neutrophil Extracellular Traps (NETs) in severe SARS-CoV-2 Lung Disease. Int J Mol Sci.

[66] Capra M, Nuciforo PG, Confalonieri S, Quarto M, Bianchi M, Nebuloni M, Boldorini R, Pallotti F, Viale G, Gishizky ML (2006). Frequent alterations in the expression of serine/threonine kinases in human cancers. Cancer Res.

[67] Wu KE, Fazal FM, Parker KR, Zou J, Chang HY (2020). RNA-GPS predicts SARS-CoV-2 RNA residency to host mitochondria and Nucleolus. Cell Syst.

[68] Pérez-Pulido AJ, Asencio-Cortés G, Brokate-Llanos AM, Brea-Calvo G, Rodríguez-Griñolo R, Garzón A, Muñoz MJ (2021). Serial co-expression analysis of host factors from SARS-CoV viruses highly converges with former high-throughput screenings and proposes key regulators. Brief Bioinform.

[69] Bukreyeva N, Sattler R, Mantlo E, Wanninger T, Manning J, Huang C, Paessler S, Zeldis J (2020). The IMPDH inhibitor merimepodib provided in combination with the adenosine analogue remdesivir reduces SARS-CoV-2 replication to undetectable levels in vitro. F1000Res.

[70] Torres B, Alcubilla P, González-Cordón A, Inciarte A, Chumbita M, Cardozo C, Meira F, Giménez M, de Hollanda A, Soriano A (2021). Impact of low serum calcium at hospital admission on SARS-CoV-2 infection outcome. Int J Infect Dis.

[71] Alemzadeh E, Alemzadeh E, Ziaee M, Abedi A, Salehiniya H (2021). The effect of low serum calcium level on the severity and mortality of Covid patients: a systematic review and meta-analysis. Immun Inflamm Dis.

[72] Pechlivanidou E, Vlachakis D, Tsarouhas K, Panidis D, Tsitsimpikou C, Darviri C, Kouretas D, Bacopoulou F (2022). The prognostic role of micronutrient status and supplements in COVID-19 outcomes: a systematic review. Food Chem Toxicol.

[73] Hu B, Huang S, Yin L (2021). The cytokine storm and COVID-19. J Med Virol.

[74] Jiang Y, Rubin L, Peng T, Liu L, Xing X, Lazarovici P, Zheng W (2022). Cytokine storm in COVID-19: from viral infection to immune responses, diagnosis and therapy. Int J Biol Sci.

[75] Zhang X, Pan C, Wei X, Yu M, Liu S, An J, Yang J, Wei B, Hao W, Yao Y (2023). Cancer-keeper genes as therapeutic targets. iScience.

[76] Wierbowski SD, Liang S, Liu Y, Chen Y, Gupta S, Andre NM, Lipkin SM, Whittaker GR, Yu H (2021). A 3D structural SARS-CoV-2–human interactome to explore genetic and drug perturbations. Nat Methods.

[77] Zhang X, Lv T, Yang X, Zhang B (2014). Structural controllability of complex networks based on preferential matching. PLoS ONE.

[78] Ruths J, Ruths D (2014). Control profiles of complex networks. Science.

[79] Zhang X, Lv T, Pu Y (2016). Input graph: the hidden geometry in controlling complex networks. Sci Rep.

[80] Zhang X, Han J, Zhang W (2017). An efficient algorithm for finding all possible input nodes for controlling complex networks. Sci Rep.

[81] Saramäki J, Kivelä M, Onnela J-P, Kaski K, Kertesz J (2007). Generalizations of the clustering coefficient to weighted complex networks. Phys Rev E.

[82] Langville AN, Meyer CD (2005). A survey of eigenvector methods for web information retrieval. SIAM Rev.

[83] Page L, Brin S, Motwani R, Winograd T. The PageRank citation ranking: Bringing order to the web. (Stanford InfoLab, 1999).

[84] Hagberg A, Swart PJ, Schult DA. Exploring network structure, dynamics, and function using NetworkX. *Conference: SCIPY 08; August 21, 2008 ; Pasadena, Pasadena, CA (United States), 21 Aug 2008*Edition edn Medium: ED (2008 Published).

[85] Zhou Y, Zhou B, Pache L, Chang M, Khodabakhshi AH, Tanaseichuk O, Benner C, Chanda SK (2019). Metascape provides a biologist-oriented resource for the analysis of systems-level datasets. Nat Commun.

