# Supplementary material for: Total network controllability analysis discovers explainable drugs for Covid-19 treatment
Source: Biol Direct. 2023 Sep 5;18:55. doi: 10.1186/s13062-023-00410-9 (PMC10478273; doi:10.1186/s13062-023-00410-9)
Supplement: Supplementary file 1 — Supplementary Material 1 [file 13062_2023_410_MOESM1_ESM.pdf]

Supplementary Materials for

Total network controllability analysis discovers explainable drugs for Covid-19 treatment

Xinru Wei+, Chunyu Pan+, Xizhe Zhang*, Weixiong Zhang*

*Corresponding author: **WZ:**weixiong.zhang@polyu.edu.hk; **XZ**: zhangxizhe@njmu.edu.cn

This file includes:

Result S1. Control hubs as drug targets for Covid-19 treatment

Method S1. Node ranking methods

Method S2. Enrichment analysis

Method S3. Identifying control hubs of complex networks

Fig. S1. The enrichment of control hubs and druggable control hubs within the k-step community.

Fig. S2. Network topologies of 22 SARS-Cov-2 proteins and 65 druggable control hubs within the 2-step community.

Fig. S3. The biological-process enrichment of the 612 non-druggable control hubs within the 2-step community.

Legends for tables S1 to S5

Other Supplementary Materials for this manuscript include:

Tables S1 to S5

Result S1. Control hubs as drug targets for Covid-19 treatment

SLC10A1 and SLC10A6 are members of the family of sodium/bile acid cotransporters, also known as Na^+-dependent taurocholate co-transporting polypeptides (NTCP). Besides the involvement in cholesterol homeostasis, SLC10A1, the founding member of the SLC10A family, takes part in HBV and HDV infections as a receptor of viral entry[1]. Such antiviral activities allude to its potential function in SARS-CoV-2 infection. SLC10A1 is targeted by 18 drugs, among which 9 have entered clinical trials for Covid-19 treatment, and SLC10A6 by two drugs. Four of these 18 drugs targeting SLC10A1 are worth mentioning. The first is Conjugated estrogens, which also target COMT, as discussed earlier. The second is Progesterone, another type of female hormone (Progestin), which can boost innate inflammatory responses. It is effective in reducing the severity of Covid-19 in pilot clinical studies[2,3]. The third is Indomethacin, a non-steroidal anti-inflammatory drug with functions of antitumor activities and antiviral activities against hepatitis B virus, rhabdovirus, and vesicular stomatitis virus. Importantly, it has been shown to relieve symptom severities and maintain oxygen saturation levels in Covid-19 patients[4,5]. The fourth is Cyclosporine A (CsA), an immunosuppressive drug widely used in the prevention of graft rejection in organ transplants. A few *in vitro* studies demonstrated the antiviral function of CsA against SARS-CoV-2 replication[6-8], and several clinical trials are currently underway[9]. Interestingly, SLC10A6 is targeted by two drugs that are distinct from the ones targeting SLC10A1. The first drug, Pregnenolone, is a hormone naturally produced in the human adrenal gland or from cholesterol and is a precursor to many other hormones including Progesterone, Estrogen, and dehydroepiandrosterone (DHEA)[10]. While Pregnenolone has been often used in treating neuropsychiatric disorders, its potential function in Covid-19 is due to its emerging anti-inflammatory role in repressing the Toll-Like Receptor 4 (TLR4) signaling pathway[11] which plays an important part in initiating the innate immune response[12]. The second drug targeting SLC10A6 is Prasterone sulfate or dehydroepiandrosterone sulfate (DHEA-S), which is a natural androstane steroid released from the adrenal gland and used as a labor inducer in childbirth. DHEA-S has been indicted in inflammatory diseases[10] and extremely low levels of DHEA-S have been detected in patients with septic shock due to cytokine storms[13]. Remarkably, DHEA-S and DHEA levels are inversely correlated with the abundance of serum interleukin-6 (IL-6)[14], whereas Covid-19 patients have a significantly elevated amount of proinflammatory cytokines, especially IL-6[15,16]. In short, the large number of drugs targeting the two members of the NTCP family attempt to boost essential hormones to enhance immunity against SARS-CoV-2 infection and suppress excessive proinflammatory cytokines that may potentially adversely cause organ damage.

MUC1 encodes a transmembrane protein in the mucin family that plays an essential role in forming protective mucous barriers on mucosal epithelial cell surfaces in various tissues and organs, including the lung, stomach, and pancreas[17]. An elevated abundance of MUC1 is indicative of the development of acute lung injury (ALI) and acute respiratory distress syndrome (ARDS)[18], which are symptoms of Covid-19 patients[19,20]. MUC1 is targeted by Potassium nitrate, an ingredient in toothpaste to alleviate tooth sensitivity to temperature and acids. A small clinical trial with 5 patients showed that the medicine helps bring the levels of oxygen saturation to above baselines in Covid-19 patients[21]. While MUC1 does not seem to be a direct target of Fostamatinib, which targets ten control hubs including RIPK1 (Tables 2, S5), it has been shown that Fostamatinib can reduce MUC1 expression as a repurposed drug for Covid-19[22].

TTPA is a protein that binds α-tocopherol, a form of vitamin E, and regulates vitamin E levels by transporting vitamin E between membrane vesicles and facilitating vitamin E secretion from hepatocytes to circulating lipoproteins. Clinical studies reveal that the baseline plasma levels of α-tocopherol are lower than normal in patients with ARDS[23] and vitamin E is beneficial for relieving the burden of upper respiratory tract infections[24]. Therefore, it is not unexpected that TTPA is the target of six vitamin E supplements, which have been recommended as adjuvant therapy against SARS-CoV-2 infection[25,26].

Note that the 2-step community also hosted 612 control hubs that were not targets of the existing drugs (Table S4B). We postulated that they were ideal candidate targets for new drugs. It was encouraging that these control hubs were enriched with membrane proteins and proteins functioning on the NF-κB signaling pathway (Figure S3), so the result invited further investigation for new drug discovery.

Method S1. Node ranking methods

Several methods are available for ranking nodes based on their connectivity and network topologies. In the sequel, a network with n nodes is considered. The degree centrality[27] of node i, $\mathrm{dc}_i = \frac{degree_i}{n-1}$, is determined by the number of neighbors connected to the node. The average neighbor degree[28] is defined as $\mathrm{nd}_i = \frac{\sum_{j \in N(i)} k_j}{|N(i)|}$, where $N(i)$ are the neighbors of node i and k_j is the degree of node j. The betweenness centrality[29] is $\mathrm{bc}_i = \sum_{s \neq i \neq t \in n} \frac{p_{s,t}(i)}{p_{s,t}}$, where $p_{s,t}(i)$ is the number of shortest paths between nodes s and t going through node i, and $p_{s,t}$ is the number of all shortest paths between nodes s and t. The load centrality is slightly different from the betweenness centrality and can be calculated according to a method from newman[30,31]. The closeness centrality[32] is written as $\mathrm{cc}_i = \frac{r-1}{n-1} \frac{r-1}{\sum_{j=1}^{r-1} d_{i,j}}$, where $d_{i,j}$ is the length of the shortest path between nodes i and j, and r is the number of nodes reachable from node i. The Eigenvector centrality[33,34] measures the importance of a node based on the centrality of its neighbors. At convergence, it can be expressed by $\mathrm{Ax} = \lambda \mathrm{x}$, where A is the network adjacency matrix with eigenvalue λ. The clustering coefficient[35] $cl_i = \frac{2T_i}{d_i(d_i-1)}$, where T_i is the number of triangles including node i and d_i is the degree of node i. The K-core[36] of a node corresponds to the largest subnet with node degree *k* or greater. The core value of a node is the largest value k containing the node. Page rank[37,38] is a node ranking method based on network structures and is used to measure the importance of a web page relative to the other pages. All these ranking methods are available in networkx[39] and coded in python.

Method S2. Enrichment analysis

Let *U* be the universe and *F* the set with a feature of interest. We were interested in the enrichment of the feature for a given set *D*. For example, consider all proteins in the human PPI network that were no more than 2-steps away from viral proteins (i.e., the universe *U*) and the subset of these proteins that were drug targets (i.e., set *F* with the feature). We were interested in the enrichment of drug targets (i.e., the feature) for the control hubs (i.e., the given set *D*). The feature enrichment of *D* can be computed as $D_F = D \cap F$. To assess the enrichment significance for D_F, a series of random sampling and a statistical test were carried out. A random sample *S* was generated by randomly drawing $|D|$ items (proteins) from *U*. $S_F = S \cap F$ was the subset of *S* with the feature of interest and a measure of feature enrichment for *S*. An empirical distribution of S_F can be derived from multiple random samplings of *S*. This empirical distribution can be taken as a baseline enrichment of the feature for items in *U*. A z-test was then adopted to evaluate the difference and significance between D and the baseline S_F . The z-test, modeled as a two-tailed Gaussian distribution, was conducted based on $Z = \frac{D_F - mean(S_F)}{SD\ of\ S_F}$, where $SD\ of\ S_F$ was the standard deviation of S_F from, say 1,000, samples. The significance of the enrichment of D_F is quantified by the *p*-value, which was from the standard normal distribution cumulative probability table.

The difference between an empirical normal distribution of S_F and another empirical normal distribution of $S_F{}'$ was analyzed using Pearson's Chi-squared test, as $\chi^2 = \sum_{i=1}^{k} \frac{(f_i - np_i)^2}{np_i}$, where k was the number of intervals; np_i was the frequency of interval i in theoretical observed distribution np (i.e., the frequency of baseline distribution S_F); f_i was the frequency of interval i in observed distribution f (as the frequency of enrichment distribution of all driver nodes). The final difference χ^2-value was calculated, and the significance *p*-value was from the Chi-square distribution table.

Method S3. Identifying control hubs of complex networks

Based on structural controllability theory, for a directed network G(V, E), the matching edges of a maximum matching form the cactus structures in the network, which are the basic control structure of the network. Therefore, the matched edges form a set of edge-independence paths in the directed network $G(V, E)$, we call these paths as ***control paths***[40]. The control paths start with driver nodes and end with tail nodes. The driver nodes (unmatched nodes) and the corresponding control path in the network are called a ***control scheme***. Such a node always remains as a middle node of a control path in all control schemes and thus is referred to as a ***control hub***. An eminent feature of a control hub is that it is essential for controlling the network regardless of which control scheme is applied to the network. A perturbation to any control hub may make the network uncontrollable by any control scheme. Therefore, it is critically important to protect all control hubs to maintain structural controllability.

So, we need to find all control hubs. This seemed to require computing all control schemes, which is #P-hard[41]. We developed an efficient algorithm without computing all control schemes. The process for identification of all control hubs is as follows[42]:

ALGORITHM: Identifying Control Hub

```
Input: bipartite graph B(V_in, V_out, E);
Output: control hub set C;
// finding all possible head nodes
initial all possible head set H;
Repeat
    Find all alternating paths AP from all unmatched nodes based on the matching M from B(V_in, V_out, E);
    H=AP∈V_in;
    If hasAugmentingPaths(AP) then
        Set M=M' obtained by expanding augmenting paths;
        Clear H;
    End
Until no augmenting path
// finding all possible tail nodes
Get transpose graph B'(V_out, V_in, E) from B(V_in, V_out, E);
initial Matching M
initial all possible tail set T;
Repeat
    Find all alternating paths AP from all unmatched nodes based on the matching M from B'(V_out, V_in, E);
    T=AP∈V_out;
    If hasAugmentingPaths(AP) then
        Set M=M' obtained by expanding augmenting paths;
        Clear T;
    End
Until no augmenting path
// finding control hub
Find all control hub C=V-H-T
```

Supplemental Reference

1 Yan, H. *et al.* Sodium taurocholate cotransporting polypeptide is a functional receptor for human hepatitis B and D virus. *Elife* **1**, e00049, doi:10.7554/eLife.00049 (2012).

2 Ghandehari, S. *et al.* Progesterone in Addition to Standard of Care vs Standard of Care Alone in the Treatment of Men Hospitalized With Moderate to Severe COVID-19: A Randomized, Controlled Pilot Trial. *Chest* **160**, 74-84, doi:10.1016/j.chest.2021.02.024 (2021).

3 Shah, S. B. COVID-19 and Progesterone: Part 1. SARS-CoV-2, Progesterone and its potential clinical use. *Endocr Metab Sci* **5**, 100109, doi:10.1016/j.endmts.2021.100109 (2021).

4 Ravichandran, R. *et al.* An open label randomized clinical trial of Indomethacin for mild and moderate hospitalised Covid-19 patients. *Sci Rep* **12**, 6413, doi:10.1038/s41598-022-10370-1 (2022).

5 Shekhar, N., Kaur, H., Sarma, P., Prakash, A. & Medhi, B. Indomethacin: an exploratory study of antiviral mechanism and host-pathogen interaction in COVID-19. *Expert Rev Anti Infect Ther* **20**, 383-390, doi:10.1080/14787210.2022.1990756 (2022).

6 Pizzorno, A. *et al.* In vitro evaluation of antiviral activity of single and combined repurposable drugs against SARS-CoV-2. *Antiviral Res* **181**, 104878, doi:10.1016/j.antiviral.2020.104878 (2020).

7 Softic, L. *et al.* Inhibition of SARS-CoV-2 Infection by the Cyclophilin Inhibitor Alisporivir (Debio 025). *Antimicrob Agents Chemother* **64**, doi:10.1128/aac.00876-20 (2020).

8 Ogando, N. S. *et al.* SARS-coronavirus-2 replication in Vero E6 cells: replication kinetics, rapid adaptation and cytopathology. *J Gen Virol* **101**, 925-940, doi:10.1099/jgv.0.001453 (2020).

9 Devaux, C. A., Melenotte, C., Piercecchi-Marti, M. D., Delteil, C. & Raoult, D. Cyclosporin A: A Repurposable Drug in the Treatment of COVID-19? *Front Med (Lausanne)* **8**, 663708, doi:10.3389/fmed.2021.663708 (2021).

10 Tomo, S., Banerjee, M., Sharma, P. & Garg, M. Does dehydroepiandrosterone sulfate have a role in COVID-19 prognosis and treatment? *Endocr Regul* **55**, 174-181, doi:10.2478/enr-2021-0019 (2021).

11 Pinna, G. Sex and COVID-19: A Protective Role for Reproductive Steroids. *Trends Endocrinol Metab* **32**, 3-6, doi:10.1016/j.tem.2020.11.004 (2021).

12 Kuzmich, N. N. *et al.* TLR4 Signaling Pathway Modulators as Potential Therapeutics in Inflammation and Sepsis. *Vaccines (Basel)* **5**, doi:10.3390/vaccines5040034 (2017).

13 Beishuizen, A., Thijs, L. G. & Vermes, I. Decreased levels of dehydroepiandrosterone sulphate in severe critical illness: a sign of exhausted adrenal reserve? *Crit Care* **6**, 434-438, doi:10.1186/cc1530 (2002).

14 Cutolo, M., Foppiani, L. & Minuto, F. Hypothalamic-pituitary-adrenal axis impairment in the pathogenesis of rheumatoid arthritis and polymyalgia rheumatica. *J Endocrinol Invest* **25**, 19-23 (2002).

15 Chen, G. *et al.* Clinical and immunological features of severe and moderate coronavirus disease 2019. *J Clin Invest* **130**, 2620-2629, doi:10.1172/jci137244 (2020).

16 Huang, C. *et al.* Clinical features of patients infected with 2019 novel coronavirus in Wuhan, China. *Lancet* **395**, 497-506, doi:10.1016/s0140-6736(20)30183-5 (2020).

17 Kato, K., Lillehoj, E. P., Lu, W. & Kim, K. C. MUC1: The First Respiratory Mucin with an Anti-Inflammatory Function. *J Clin Med* **6**, doi:10.3390/jcm6120110 (2017).

18 Nakashima, T. *et al.* Circulating KL-6/MUC1 as an independent predictor for disseminated intravascular coagulation in acute respiratory distress syndrome. *J Intern Med* **263**, 432-439, doi:10.1111/j.1365-2796.2008.01929.x (2008).

19 Ruan, Q., Yang, K., Wang, W., Jiang, L. & Song, J. Clinical predictors of mortality due to COVID-19 based on an analysis of data of 150 patients from Wuhan, China. *Intensive Care Med* **46**, 846-848, doi:10.1007/s00134-020-05991-x (2020).

20 Zhou, F. *et al.* Clinical course and risk factors for mortality of adult inpatients with COVID-19 in Wuhan, China: a retrospective cohort study. *Lancet* **395**, 1054-1062, doi:10.1016/s0140-6736(20)30566-3 (2020).

21 Ostojic, S. M., Milovancev, A., Drid, P. & Nikolaidis, A. Oxygen saturation improved with nitrate-based nutritional formula in patients with COVID-19. *J Int Med Res* **49**, 3000605211012380, doi:10.1177/03000605211012380 (2021).

22 Kost-Alimova, M. *et al.* A High-Content Screen for Mucin-1-Reducing Compounds Identifies Fostamatinib as a Candidate for Rapid Repurposing for Acute Lung Injury. *Cell Rep Med* **1**, 100137, doi:10.1016/j.xcrm.2020.100137 (2020).

23 Jovic, T. H. *et al.* Could Vitamins Help in the Fight Against COVID-19? *Nutrients* **12**, doi:10.3390/nu12092550 (2020).

24 Meydani, S. N. *et al.* Vitamin E and respiratory tract infections in elderly nursing home residents: a randomized controlled trial. *Jama* **292**, 828-836, doi:10.1001/jama.292.7.828 (2004).

25 Samad, N. *et al.* Fat-Soluble Vitamins and the Current Global Pandemic of COVID-19: Evidence-Based Efficacy from Literature Review. *J Inflamm Res* **14**, 2091-2110, doi:10.2147/jir.S307333 (2021).

26 Beigmohammadi, M. T. *et al.* The effect of supplementation with vitamins A, B, C, D, and E on disease severity and inflammatory responses in patients with COVID-19: a randomized clinical trial. *Trials* **22**, 802, doi:10.1186/s13063-021-05795-4 (2021).

27 Borgatti, S. P. & Halgin, D. S. Analyzing affiliation networks. *The Sage handbook of social network analysis* **1**, 417-433 (2011).

28 Barrat, A., Barthelemy, M., Pastor-Satorras, R. & Vespignani, A. The architecture of complex weighted networks. *Proceedings of the national academy of sciences* **101**, 3747-3752 (2004).

29 Brandes, U. On variants of shortest-path betweenness centrality and their generic computation. *Social networks* **30**, 136-145 (2008).

30 Newman, M. E. Scientific collaboration networks. II. Shortest paths, weighted networks, and centrality. *Physical review E* **64**, 016132 (2001).

31 Goh, K.-I., Kahng, B. & Kim, D. Universal behavior of load distribution in scale-free networks. *Physical review letters* **87**, 278701 (2001).

32 Freeman, L. Centrality in networks: I. conceptual clarifications. social networks. *Social Network* (1979).

33 Bonacich, P. Power and centrality: A family of measures. *American journal of sociology* **92**, 1170-1182 (1987).

34 Brede, M. *Networks—An Introduction. Mark EJ Newman.(2010, Oxford University Press.) $65.38,£ 35.96 (hardcover), 772 pages. ISBN-978-0-19-920665-0.* (MIT Press One Rogers Street, Cambridge, MA 02142-1209, USA journals-info …, 2012).

35 Saramäki, J., Kivelä, M., Onnela, J.-P., Kaski, K. & Kertesz, J. Generalizations of the clustering coefficient to weighted complex networks. *Physical Review E* **75**, 027105 (2007).

36 Batagelj, V. & Zaversnik, M. An O (m) algorithm for cores decomposition of networks. *arXiv preprint cs/0310049* (2003).

37 Langville, A. N. & Meyer, C. D. A survey of eigenvector methods for web information retrieval. *SIAM review* **47**, 135-161 (2005).

38 Page, L., Brin, S., Motwani, R. & Winograd, T. The PageRank citation ranking: Bringing order to the web. (Stanford InfoLab, 1999).

39 Hagberg, A., Swart, P. & S Chult, D. Exploring network structure, dynamics, and function using NetworkX. (Los Alamos National Lab.(LANL), Los Alamos, NM (United States), 2008).

40 Ruths, J. & Ruths, D. Control profiles of complex networks. *Science* **343**, 1373-1376 (2014).

41 Valiant, Leslie G. The complexity of computing the permanent. *Theoretical computer science* **8.2** (1979): 189-201.

42 Zhang, X., Pan, C. & Zhang, W. Control hubs of complex networks and a polynomial-time identification algorithm. arXiv:2206.01188 (2022). doi: 10.48550/arXiv.2206.01188.

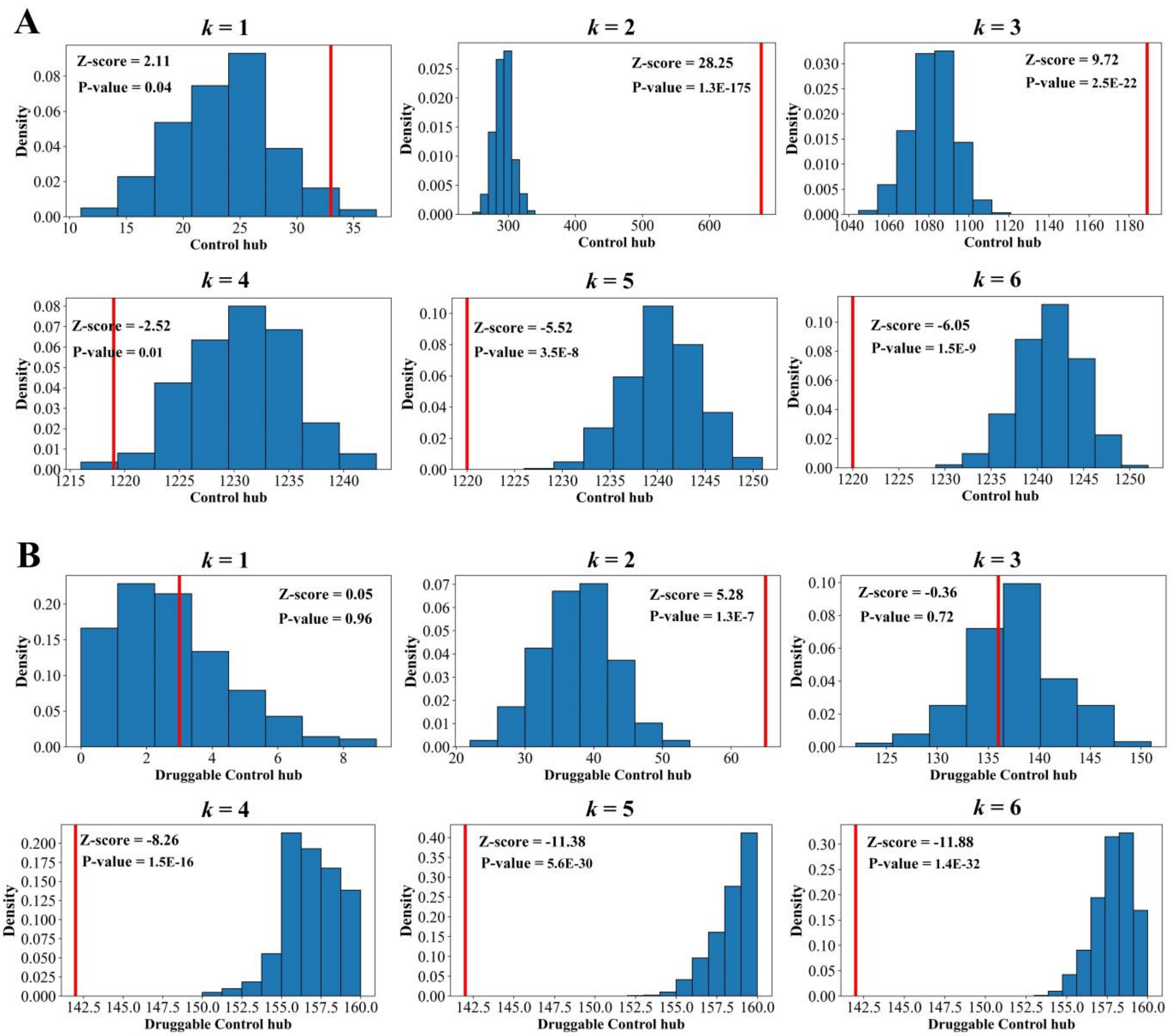

Fig. S1. The enrichment of control hubs and druggable control hubs within the k-step community. Identification of the community of human proteins *k*-steps away from SARS-CoV-2 proteins which are enriched with control hubs and druggable control hubs. A) A statistical analysis, a series z-test, was adopted to compare the number of control hubs within a k-step community against a random empirical distribution (see Methods). The numbers of control hubs within the communities are shown in red lines, and their respective baseline random distributions are shown in blue bars. The 2-step community was enriched with control hubs. **B)** Similarly, the 2-step community was enriched with druggable control hubs.

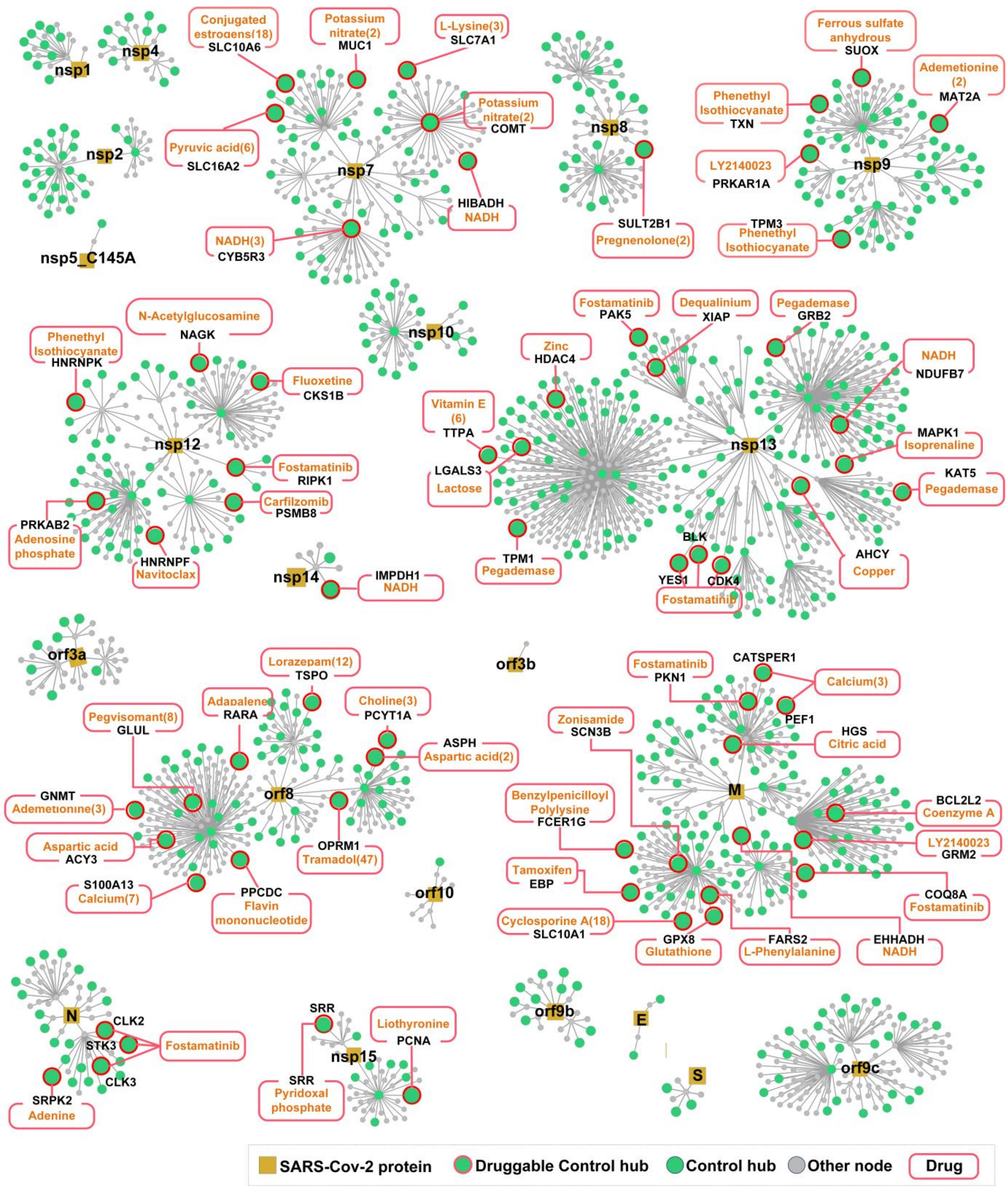

Fig. S2. Network topologies of 22 SARS-Cov-2 proteins and 65 druggable control hubs within the 2-step community. The major drugs and the number of drugs targeting the control hubs are shown in red boxes, only one drug is shown for each control hub. Detailed information is available in Table S4A.

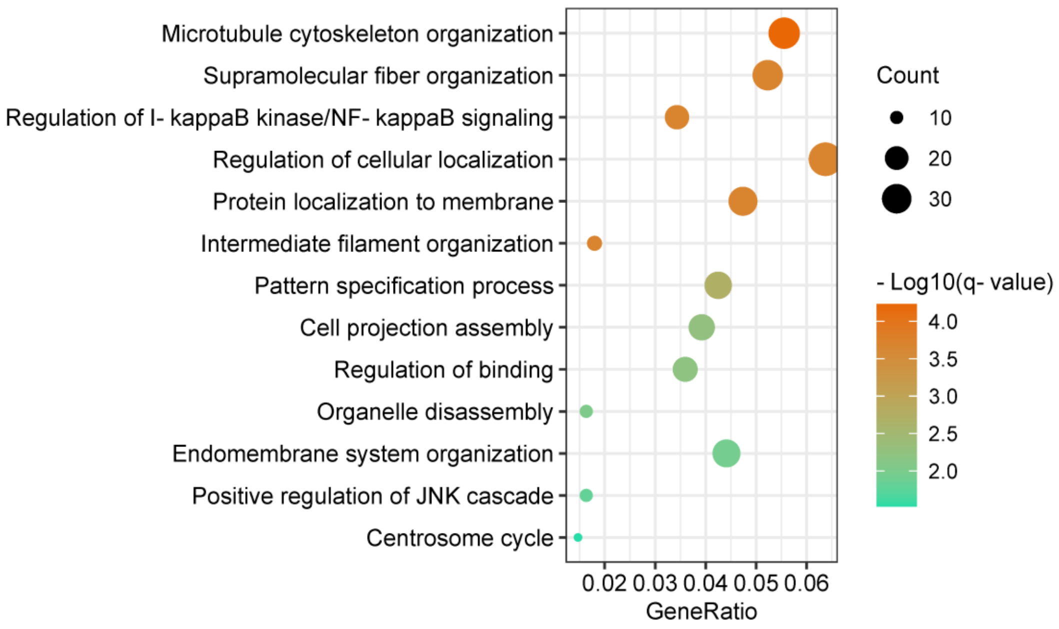

Fig. S3. The biological-process enrichment of the 612 non-druggable control hubs within the 2-step community, revealing their collective functions during viral infection. GeneRatio is the ratio between the number of observed proteins with a specific Go term and the total number of proteins of interest.

Captions for Tables S1-S5, which are the individual tabs in a single Excel spreadsheet file that includes the raw data or results. We also put them in GitHub (https://github.com/network-control-lab/control-hubs).

Table S1: Human 9,092 proteins and 64,006 interactions in Huri-Union PPI. We constructed a triple-layer network of PPIs between humans and SARS-CoV-2 and within humans as well as interactions between drugs and human target proteins. The middle layer of the triple-layer network comes from the Huri-Union database, which consists of 9,092 proteins or nodes (in Table S1A) and 64,004 interactions or edges (in Table S1B).

Table S2: 169 PPIs between human and SARS-CoV-2 proteins. 169 high-confidence virus-host interactions reported by Gordon et al. are used to establish our triple-layer network, which consists of 22 SARS-CoV-2 proteins and 169 host proteins.

Table S3: Drugs and their human protein targets. The drug-target interactions (17,780 interactions between 2,913 targets and 2,979 drugs) collected from the Drugbank database are regarded as the third layer of the triple-layer network. The drugs in this network are FDA-approved or under clinical investigation for the treatment of Covid-19.

Table S4: 677 Control hubs in the human PPI network which are no more than two steps away from a SARS-Cov-2 protein. Among the 677 control hubs, 65 are druggable control hubs targeted by at least one drug (in Table S4A), and the rest 612 control hubs (in Table S4B) are potential targets for new drugs.

Table S5: 185 candidate drugs for Covid-19 therapy and prevention. A total of 185 candidate drugs are identified from FDA-approved or investigated drugs targeting 65 druggable control hubs.
